# Kidney disease in adults with Prader-Willi syndrome: international cohort study and systematic literature review

**DOI:** 10.3389/fendo.2023.1168648

**Published:** 2023-07-21

**Authors:** Denise H. van Abswoude, Karlijn Pellikaan, Naomi Nguyen, Anna G. W. Rosenberg, Kirsten Davidse, Franciska M. E. Hoekstra, Ilse M. Rood, Christine Poitou, Graziano Grugni, Charlotte Høybye, Tania P. Markovic, Assumpta Caixàs, Antonino Crinò, Sjoerd A. A. van den Berg, Aart J. van der Lely, Laura C. G. de Graaff

**Affiliations:** ^1^ Department of Internal Medicine, Division of Endocrinology, Erasmus Medical Center, University Medical Center Rotterdam, Rotterdam, Netherlands; ^2^ Center for Adults with Rare Genetic Syndromes, Department of Internal Medicine, Division of Endocrinology, Erasmus Medical Center, University Medical Center Rotterdam, Rotterdam, Netherlands; ^3^ Dutch Center of Reference for Prader–Willi Syndrome, Rotterdam, Netherlands; ^4^ Academic Center for Growth Disorders, Erasmus Medical Center, University Medical Center Rotterdam, Rotterdam, Netherlands; ^5^ Internal Medicine, Division of Nephrology, Reinier de Graaf Gasthuis, Delft, Netherlands; ^6^ Department of Nephrology, Radboud University Medical Center, Radboud Institute for Health Sciences, Nijmegen, Netherlands; ^7^ Assistance Publique-Hôpitaux de Paris, Rare Diseases Center of Reference ‘Prader-Willi Syndrome and Obesity with Eating Disorders’ (PRADORT), Nutrition Department, Institute of Cardiometabolism and Nutrition (ICAN), Pitié-Salpêtrière Hospital, Sorbonne Université, National Institute of Health and Medical Research (INSERM), Nutriomics, Paris, France; ^8^ International Network for Research, Management & Education on adults with Prader-Willi Syndrome (INfoRMEd-PWS); ^9^ European Reference Network on Rare Endocrine Conditions (ENDO-ERN); ^10^ Division of Auxology, Istituto Auxologico Italiano, Istituto di Ricovero e Cura a Carattere Scientifico (IRCCS), Piancavallo, Italy; ^11^ Department of Molecular Medicine and Surgery, Karolinska Institute and Karolinska University Hospital, Stockholm, Sweden; ^12^ Department of Endocrinology, Karolinska Institute and Karolinska University Hospital, Stockholm, Sweden; ^13^ Metabolism & Obesity Service, Royal Prince Alfred Hospital, Camperdown, NSW, Australia; ^14^ Charles Perkins Center and Sydney Medical School, University of Sydney, Sydney, NSW, Australia; ^15^ Department of Endocrinology and Nutrition, Parc Tauli Hospital Universitari, Institut d’Investigació i Innovació Parc Taulí (I3PT) Instituto de Salud Carlos III (CERCA-ISCIII), Sabadell, Spain; ^16^ Department of Medicine, Universitat Autònoma de Barcelona, Sabadell, Spain; ^17^ Reference Center for Prader-Willi syndrome, Bambino Gesù Hospital, Research Institute, Palidoro, Italy; ^18^ Center for Rare Diseases and Congenital Defects, Fondazione Policlinico Universitario A. Gemelli, Istituto di Ricovero e Cura a Carattere Scientifico (IRCCS), Rome, Italy; ^19^ Department of Clinical Chemistry, Erasmus Medical Center (MC), University Medical Center Rotterdam, Rotterdam, Netherlands

**Keywords:** Prader-Willi Syndrome, kidney function tests, proteinuria, urine tract infections, cardiovascular disease, kidney disease

## Abstract

**Background:**

Prader-Willi syndrome (PWS) is a rare, complex, genetic disorder characterized by hyperphagia, hypotonia, delayed psychomotor development, low muscle mass and hypothalamic dysfunction. Adults with PWS often have obesity, hypertension and type 2 diabetes mellitus (DM2), known risk factors for cardiovascular disease (CVD) and chronic kidney disease (CKD). Early symptoms of CVD and CKD may be masked by intellectual disability and inability to express physical complaints. Furthermore, kidney diseases are often asymptomatic. Therefore, renal and cardiovascular disease might be missed in patients with PWS. Microalbuminuria is an early sign of microvascular damage in the kidneys and other vascular beds. Therefore, we screened our adult PWS cohort for the presence of elevated urinary albumin and (micro)albuminuria.

**Methods:**

We retrospectively collected anthropometric measurements, blood pressure, medical history, medication use, urine dipstick and biochemical measurements form electronic patient files. In addition, we performed a systematic literature review on kidney disease in PWS.

**Results:**

We included 162 adults with genetically confirmed PWS (56% male, median age 28 years), of whom 44 (27%) had DM2. None had known CVD. All subjects had normal estimated glomerular filtration rate (eGFR) according to non-PWS reference intervals. Elevated urinary albumin or (micro)albuminuria was present in 28 (18%); 19 out of 75 (25%) had an increased urinary albumin-to-creatinine ratio (UACR) and 10 out of 57 (18%) had an increased urinary protein-to-creatinine ratio. Elevated urinary albumin was present at a young age (median age 26 (IQR 24-32) years) and was associated with an significantly higher BMI and LDL-cholesterol levels and higher prevalence of DM2, hypertension and dyslipidemia than those with normal UACR (*p*=0.027, *p*=0.019, *p*<0.001, *p*<0.001, *p*=0.011 and respectively).

**Conclusion:**

Upon screening, one in every five adults with PWS had increased urinary albumin or (micro)albuminuria, early signs of microvascular disease. All had normal eGFR, according to non-PWS reference intervals, and none had a formal diagnosis of CVD. As muscle mass is low in PWS, creatinine levels and eGFR may be spuriously normal. Urinalysis in this patient group can be used as a screening tool for microvascular (kidney) disease. We propose an algorithm for the detection and management of microvascular disease in adults with PWS.

## Introduction

Prader-Willi syndrome (PWS) is a complex, genetic, neurodevelopmental disorder caused by loss of expression of a cluster of paternally expressed genes on chromosome 15q11-13 (1). In most cases, this syndrome is caused by a paternal deletion (60-70%) or a maternal uniparental disomy (mUPD, 25-35%). Less common genetic findings include imprinting center defects (ICD, 1-3%), balanced translocations (0.1%) and gene mutations (<0.1%) (1, 2).

During early infancy, PWS is characterized by hypotonia and failure to thrive due to a poor sucking reflex and lethargy (1, 3, 4). In childhood, motor development is delayed and adults often show muscle weakness and decreased muscle mass (5–7).In both children and adults, hypothalamic dysfunction often results in pituitary hormone deficiencies, such as hypogonadism (estimated in 98% in males and 94% in females), growth hormone deficiency and hypothyroidism (estimated prevalence of 14-17%) (1, 8–14). Especially in patients without growth hormone treatment (GHt) (1, 15–19), basal metabolic rate (BMR) is low due to low lean body mass and reduced physical activity. The combination of low BMR and hyperphagia leads to an increased risk of obesity (42% of adults with PWS) (20). This high prevalence of obesity, in turn, leads to an increase in the prevalence of DM2 (17%), hypertension (18%) and hypercholesterolemia (19%) (20). These are known risk factors for cardiovascular disease (CVD) (20) and chronic kidney disease (CKD) (21–23).

With improved healthcare for patients with PWS, life expectancy has increased. As patients with PWS become older, age-related diseases like CVD and CKD become increasingly relevant. Early detection and treatment are crucial to prevent complications, loss of quality of life and early mortality (24, 25).

As symptoms are often atypical, non-specific or even absent (26–29), the detection of CKD can be challenging. In patients with PWS, the detection of CVD and CKD might be even more complicated due to the presence of intellectual disability (mild in the majority of patients) and reduced ability to express physical complaints (30, 31). Furthermore, patients with PWS have a high pain threshold that might mask the sudden thoracic pain or headache that may indicate cardiac or cerebrovascular events. The lack of fever and pain in people with PWS can also mask other risk factors for CKD, including chronic urinary tract infections (UTIs) (1, 8, 32, 33) that may further increase the risk of CKD (34). Although never investigated, CKD and CVD might be more difficult to detect due to the combination of physical, behavioral, and neurocognitive potentially increasing morbidity and mortality in adults with PWS.

CKD can be diagnosed based on a decreased (estimated) glomerular filtration rate (eGFR) and the presence of (micro)albuminuria (35). (Micro)albuminuria can be defined as an increased urinary albumin-to-creatinine ratio (UACR) or urinary protein-to-creatinine ratio (UPCR) and is associated with an increased mortality independent of the eGFR (36, 37). Preferably, the UACR should be assessed in first morning urine samples as it corresponds more closely with a 24-hour urine collection, which is the golden standard (35, 38, 39). Microalbuminuria can indicate microvascular injury and is therefore a risk factor for CKD and CVD (40–42). In patients with diabetes mellitus, microalbuminuria is predictive of early mortality (43). Besides (micro)albuminuria, an increase in the exertion of low molecular weight (LMW) proteins such as cystatine-C, ubiquitin, retinol binding protein or alpha1-microglobulin, might also indicate kidney disease (44–46).

Little is known about CKD and CVD in the general population of adults with PWS, as these co-morbidities have only been studied in specific ‘subtypes’ of patients with PWS. In hospitalized patients with PWS, the prevalence of CKD was estimated to be 6.5% (47). In PWS patients with DM, microalbuminuria was found in 6.9-55.6% and proteinuria in 3.4-11.1% of (48–50), which is similar to the prevalence in the general population with DM (51). Apart from these studies in PWS ‘subpopulations’ of hospitalized and/or diabetic PWS patients, microalbuminuria and proteinuria have not been systematically assessed in the broader adult PWS population. This lack of information is relevant, as both microalbuminuria and proteinuria are associated with an increased risk of CKD and cardiovascular morbidity and mortality (40–42, 52–56). Also, to our knowledge, systematic screening for kidney disease (by both urine and serum analysis) has never been performed in adults with PWS. As a result, there are no guidelines for screening and treatment of CKD in adults with PWS.

To fill this knowledge gap, we have systematically screened urine and serum samples for signs of CKD in a cohort of adults with PWS. We also assessed risk factor for CKD and performed a systematic literature review on kidney and urinary tract disease in PWS. Based on our cohort study and systematic review, we provide practical recommendations for the screening and treatment of (micro)albuminuria and CKD in patients with PWS.

## Method

This international cross-sectional study was performed at the Center for Adults with Complex Rare Genetic Syndromes (CRGS) at the Erasmus University Medical Center, Rotterdam, the Netherlands, the Rare Disease Center of reference ‘Prader-Willi Syndrome and obesity with eating disorders’, in Paris, France and the Division of Auxology in Piancavallo, Italy. Only adults with genetically confirmed PWS who visited the outpatient clinic of one of these reference center between January 2020 and December 2022 were included. Urinalysis was part of a routine systematic health screening in all included PWS patients. Ethical approval and/or individual informed consent was obtained by the Medical Ethics Committee of participating centers according to local rules and regulations.

As previously described (20), the systematic screening generally consists of a structured interview, an extensive physical examination, a medical questionnaire (depending on local guidelines), a review of medical records and biochemical measurements. The systematic screening took place during the first or follow-up visit to the outpatient clinic. If measurements could not be done during the first visit (due to behavioral or logistic issues), the next available measurement was used for the statistical analysis, provided that the time interval between first and follow up visit was less than 12 months.

Height and weight measurements were collected from medical records. When multiple measurements were available, we included the measurement the was closest (in time) to the biochemical and urinalysis. A Body Mass Index (BMI, in kg/m^2^) between 18.5 and 24.9 kg/m^2^ was considered lean, between 25.0 and 29.9 kg/m^2^ overweight, 30.0 or more kg/m^2^ obese and a BMI of 40.0 or more grade III obesity according to the World Health Organization Criteria (57).

Resting blood pressure was measured in all patients during their routine visit to the outpatient clinic. In the Dutch cohort, blood pressure was measured using a Mindray sphygmomanometer with trueBP™ technology. If the blood pressure was above 140/90 mmHg (58), the measurement was repeated. If the blood pressure was still elevated, a 30-minute automated blood pressure measurement was carried out. The most recent blood pressure was included in the analysis. As patients with PWS in the Dutch reference center were not evaluated yearly for substance abuse (smoking, alcohol), most recent data were included. However, in some cases, this was more than twelve months prior to blood and urine sample collection.

### Biochemical analysis

Blood and midstream urine samples were collected for general medical screening. As the outpatient clinic was often in the afternoon and fasting can be problematic in PWS due to hyperphagia, non-fasting blood samples were used. We evaluated kidney function (urea, creatinine, eGFR using Chronic Kidney Disease Epidemiology Collaboration (CKD-epi) calculation) and lipid profiles ((non-fasting) low density lipoprotein (LDL)-cholesterol, high density (HDL)-cholesterol, triglycerides, total cholesterol), glucose metabolism (fasting or random glucose, hemoglobin A1c (HbA1c)) in blood. In random spot urine samples, we measured microalbumin, urinary albumin-to-creatinine ratio (UACR), total protein and/or urinary protein-to-creatinine ratio (UPCR). As the outpatient clinic was in the afternoon in some centers, first morning urine samples were not available. If microalbuminuria was found, the patients were referred to the general practitioner to confirm microalbuminuria in a second urine sample. Second samples were not available for this study. A urine dipstick was used to evaluate the presence of glucose, protein, leukocytes, and nitrite. If urine and blood samples could not be collected on the same day, the blood samples with the shortest time to urinalysis were used. If urine and blood samples were collected more than 12 months apart, the blood sample was excluded from analysis.

### Cut-off levels

DM2 was defined as a fasting glucose of 7.0 mmol/L or higher (if available) or a repeated non-fasting glucose above 11.1 mmol/L. As fasting glucose were only available in a few patients, non-fasting glucoses were used in further analysis. Impaired glucose tolerance was tested using an oral glucose tolerance test and diagnosed if measured serum glucoses was between 7.8 to 11.1 mmol/L after two hours according to the American Diabetes Association classification (59). HbA1c was not used as a diagnostic criterium for DM2, but was used to monitor glycemic control. Furthermore, patients with a previous diagnosis of DM2 were considered as having DM2. Hypercholesterolemia was defined as a non-fasting LDL-cholesterol above 4.1 mmol/L and dyslipidemia was defined as a non-fasting LDL-cholesterol above 4.1 mmol/L or a (non-fasting or fasting) triglyceride above 2.0 mmol/L (60). The CKD-epi formula was used to calculate the estimated glomerular filtration rate (eGFR) values (61). An eGFR >90 mL/min was defined as normal kidney function. Microalbumin was considered elevated when >0.02 g/L (measured in one random spot urine sample). Microalbuminuria was defined as an UACR between 3 and 30 mg/mmol creatinine and proteinuria as an UACR >30 mg/mmol creatinine according to the Kidney Disease: Improving Global Outcomes (KDIGO) guideline (35). Proteinuria was defined as an UPCR of >20 mg/mmol creatinine (0.2 mg/mg) and nephrotic proteinuria as an UPCR of >300 mg/mmol creatinine (3.0 mg/mg) (62). Urinary total protein levels >0.15 g/L in a24 hour urine collection were considered elevated. An albumin excretion rate of 30-300mg/24 hours was considered increased and >300 mg/24 hours was considered severely increased or nephrotic proteinuria (35). When leukocytes and/or nitrite was positive in urine dipstick analysis, patients were referred to their general practitioner for urine culture and, if positive, (antibiotic) treatment.

### Systematic literature review

We performed a literature search in June 2020 (updated in March 2022) using Embase, Medline, the Web of Science Core Collection, Cochrane Central Register of Controlled Trials and Google Scholar (**Table S1**). Studies were assessed for eligibility using the PRISMA 2020 (63) guideline and were included if they reported on both 1) (the prevalence of) kidney disease, urological abnormalities, treatment for kidney disease or markers for kidney function and 2) PWS, Prader-Willi-like syndrome (PWLS) with an abnormality in 15q11.2-13 or one of the genes on the PWS critical region. We also scanned the references of included articles. Studies reporting on cases of kidney or urological cancer, combinations of PWS with other genetic syndromes, PWLS with a deletion extending beyond the PWS critical region and articles only describing the expression of a gene in the kidney were excluded. Meeting reports, workshop summaries, reviews, conference abstracts, guidelines, articles without full-text availability and articles that were not available in English were also excluded.

Screening based on title and abstract was performed independently by three reviewers (either NN and KP or DA and KP). Full-text screening was performed independently by DA and KP. Disagreements were resolved in a meeting with a third reviewer (LG). Data was extracted by DA and NN.

### Data analysis

Data was analyzed using IBM SPSS version 28.0. Continuous variables are presented as median [interquartile range (IQR)], dichotomous variables as number and percentage of patients n (%). To investigate the association between microalbuminuria, macroalbuminuria and the presence of risk factors (e.g. smoking, alcohol usage, DM2, blood pressure/hypertension and hypercholesterolemia), a Chi-squared test was used for dichotomous variables and a Mann-Whitney U test for continuous variables. A *p*-value of <0.05 was considered statistically significant. To adjust for BMI as possible confounder, a logistic regression analysis was performed.

## Results

We included 162 adults with PWS. Baseline characteristics are displayed in **Table 1**. Median age at urinalysis was 28 [IQR 22-38] years. Ninety out of 162 patients were male (56%). Most patients had a deletion as the underlying genotype (n=102 (63%)), followed by mUPD (n=52 (32%)) and ICD in 1%. In six patients, PWS was genetically confirmed, but the genotype was unknown or unspecified. Sixty-seven percent of patients had received GHt, 65% received GHt during childhood and 56% were currently using GHt. The median BMI was 33.0 [IQR 26.8-41.0] kg/m^2^ and 60% of subjects had obesity. Grade III obesity was present in 47 of 97 (48.5%) of patients with obesity. Forty-four (27%) patients were diagnosed with DM2 and 17out of 162 (11%) had IGT. Hypertension and hypercholesterolemia were present in 29 (18%) and 23 patients (14%) respectively. In the French cohort, dyslipidemia was present in 37%. None of the patients were diagnosed with cardiovascular events (myocardial infarction or CVA).

**Table 1:** Baseline characteristics of included adults with PWS.

	Dutch cohort *n=68*	Italian cohort *n=51*	French cohort *n=43*	Total *n=162*
**Age at urine sample, years**	29 [23-38]	34 [23-43]	21 [19-32]	28 [22-38]
**Male gender**	40 (59)	24 (47)	26 (61)	90 (56)
**Genotype** Deletion Type 1 Type 2 Atypical Unknown mUPD ICD Other Unspecified	40 (59) 8 (12) 18 (27) 6 (9) 8 (12) 24 (35) 2 (3) 2 (3) 0	40 (78) 11 (22) 0 0 0	22 (51) 17 (39) 0 2 (5) 2 (5)	102 (63) 8 (5) 18 (11) 6 (4) 70 (43) 52 (32) 2 (1) 4 (3) 2 (1)
**BMI (kg/m^2^)** Obesity^a^	28.2 [24.0-34.7] 30 (44)	35.0 [27.9-41.8] 31 (61)	40.4 [32.7-47.0] 36 (84)	33.0 [26.8-41.0] 97 (60)
**Alcohol usage** Units per week **Smoking^b^** Cigarettes per day	12 (27), *n*=44 1.2 [0.98-1.96] 6 (12), *n*=51 9 [5.3-10]	1 (2), *n=50* 7 3 (6), *n=50* 12 [10-15]	1 (9), *n=11* NA 1 (3), *n=30* NA	14 (13), *n=106* 1.5 [1.0-2.0] 10 (8), *n=131* 10 [7.5-11.5]
**Growth hormone treatment** Ever During childhood During adulthood Current	45 (66), *n=68* 41 (64), *n=64* 37 (62), *n=60* 31 (57), *n=54*	36 (71), *n=51* 28 (65), *n=43* 20 (57), *n=35* 17 (53), *n=32*	28 (65), *n*=43 NA NA NA	109 (67), *n=162* 69 (65), *n=107* 57 (60), *n=95* 48 (56), *n=86*
**Diabetes Mellitus** Type 1 Type 2 IGT DM in the past **Diabetes treatment** No medication Metformin GLP-1 Insulin only Combination without insulin Combination with insulin	0 11 (16) 7 (10) 3 (4) 1 (9), *n=11* 4 (36), *n=11* 0, *n=11* 1 (9), *n=11* 5 (46), *n=11* 0, *n=11*	0 13 (26) 10 (20) 0 1 (8), *n*=13 3 (23), *n=13* 0, *n=13* 0, *n=13* 3 (23), *n=13* 6 (46), *n*=13	0 20 (47) 0 0 0, *n=20* 1 (5), *n=20* 3 (15), *n*=20 0, *n=20* 5 (25), *n*=20 11 (55), *n=20*	0 44 (27) 17 (11) 3 (2) 2 (5), *n=44* 8 (18), *n=44* 3 (7), *n=44* 1 (2), *n=44* 13 (30), *n=44* 17 (38), *n=44*
**Pre-existing hypertension** **Hypertension treatment** ACE inhibitor ATII receptor antagonist Ca antagonist Diuretic Combination	8 (12) 7 (88), *n=8* 3 (38) 1 (13) 2 (25) 0 1 (13)	14 (28) 13 (93), *n=14* 3 (23) 0 0 2 (15) 8 (62)	7 (16) 7 (100), *n=7* 2 (29) 1 (14) 1 (14) 0 3 (43)	29 (18) 27 (93), *n=29* 8 (30) 2 (7) 3 (11) 2 (7) 12 (45)
**Hypercholesterolemia** **Dyslipidemia** Statin treatment	4 (6) NA 2 (50)	19 (37) NA 4 (21)	NA 16 (37) 8 (50)	23 (14) 16 (10) 14 (9)
**Cardiovascular event^c^**	0, *n=65*	0, *n=51*	0, *n=43*	0, *n=162*
**Laboratory results** Non fasting glucose (mmol/L) HbA1c (mmol/mol) Creatinine (µmol/L) Normal eGFR (mL/min) eGFR (mL/min/1.73m^2^)^e^ LDL-cholesterol (mmol/L)	5.2 [4.8-6.2], *n=58* 38 [35-40], *n=44* 62 [54-70], *n=60* 60 (100), *n=60* 124 [113-131], *n=60* 2.73 [2.27-3.15], *n=58*	5.0 [4.4-5.8], *n=51* 38 [33-46], *n=51* 55 [47-60], *n=51* 51 (100), *n=51* 126 [114-133], *n=51* 2.94 [2.50-3.46], *n=51*	NA^d^	5.2 [4.6-6.0], *n=109* 38 [34-43], *n=95* 58 [52-65], *n=111* 111 (100), *n=111* 125 [114-132], *n=111* 2.84 [2.33-3.18], *n*=109

Dichotomous data is displayed as number (%), continuous variables are displayed as median [IQR]. angiotensin-converting enzyme (ACE), angiotensin-II (ATII), body mass index (BMI), calcium (Ca), cholesterol (chol), diabetes mellitus (DM), glucagon-like peptide 1 agonist (GLP-1), hemoglobin A1c (HbA1c), impaired glucose tolerance (IGT), imprinting center defects (ICD), interquartile range (IQR), low-density lipoprotein (LDL), high-density lipoprotein (HDL), maternal uniparental disomy (mUPD), not available (NA). ^a^ Obesity was specified as BMI ≥30 kg/m^2^. ^b^ Three patients used to smoke in the past, but stopped at time of data collection. ^c^ Cardiovascular events included coronary disease, myocardial infarction, transient ischemic attack and stroke. ^e^ eGFR was calculated using CDK-epi. ^d^ Not calculated as dates of blood samples were unavailable.

In eight Dutch and all French patients, urine samples were present without recent blood samples (i.e. <12 months between urine and blood sample collection). Of the patients with available recent blood samples, all had normal eGFR (CKD-EPI eGFR >90 mL/min/1.73m^2^). Median eGFR (CDK-epi) was 125 [IQR 114-132] mL/min/1.73m^2^. In males, median creatinine was 61 [IQR 54-69] µmol/L (reference value 65-115 µmol/L) and eGFR was 127 [IQR 118-135] mL/min/1.73m^2^. In females, median creatinine levels were 55 [IQR 47-61] µmol/L (reference value 55-90 µmol/L) and eGFR was 120 [IQR 111-128] mL/min/1.73m^2^. All patients were classified as CKD stage G1 (35). When applying previously suggested alternative reference values for eGFR for adults with PWS (>98 ml/min/1.73m^2^ in males and >93 ml/min/1.73m^2^ in females) (20) renal function of only two males and two females were below the reference value (94 and 98 ml/min/1.73m^2^ in males and 90 and 92 ml/min/1.73m^2^ in females respectively).

### Urinalysis

#### Microalbuminuria and proteinuria

Twenty-eight out of 160 patients (18%) had elevated urine albumin, microalbuminuria or proteinuria (**Table 2**). Two patients were excluded from this analysis as data on (micro)albuminuria, proteinuria, UACR and UPCR were missing. Of the two males and two females with impaired renal function according to alternative reference values for eGFR for adults with PWS, only one female had microalbuminuria (Case 8, **Table 3**) (20).

**Table 2:** Comparison of adults with PWS and normal versus (micro)albuminuria^a^.

	Number of observations	Normal *n=132*	Number of observations	(micro)albuminuria *n=28*	p-value
**Age at urine sample (years)**	132	20 [21-38]	28	25 [23-33]	0.65
**Male gender**	132	76 (58)	28	14 (50)	0.46
**BMI (kg/m^2^)** Obesity	132	32.4 [26.4-40.4] 73 (55)	28	38.7 [31.1-43.8] 22 (79)	**0.027** **0.023**
**Genotype** Del mUPD ICD Other	132	84 (64) 42 (32) 2 (1) 4 (3)	28	16 (57) 10 (36) 0 2 (7)	0.62^b^
**Alcohol usage** Units/week **Smoking** Cigarettes/day	89 9 105 6	10 (11) 2 [1-5] 8 (8) 10 [8-13]	16 2 25 2	3 (19) NA^c^ 2 (8) NA^c^	0.40 NA^c^ 0.69 NA^c^
**GH treatment** Ever Childhood vs never Adulthood vs never Ongoing vs never	132 92 82 73	92 (70) 62 (67) 52 (63) 43 (59)	28 13 11 11	17 (61) 7 (54) 5 (46) 5 (46)	0.35 0.34 0.25 0.40
**Diabetes Mellitus type 2**	132	28 (21)	28	16 (57)	**<0.001^d^**
**Pre-existing hypertension**	132	17 (13)	28	11 (39)	**<0.001^e^**
**Blood pressure** Systolic Diastolic	128	125 [119-133] 80 [70-83]	26	124 [117-141] 80 [70-88]	0.49 0.65
**Hypercholesterolemia** **Dyslipidemia**	103 29	20 (19) 7 (24)	14 14	3 (21) 9 (64)	0.86 **0.011^f^**
**Laboratory results** Glucose (mmol/L) HbA1c (mmol/mol) Creatinine (umol/L) eGFR (mL/min/1.73m^2^) LDL cholesterol (mmol/L)	97 84 98 98 97	5.1 [4.6-6.0] 38 [34-42] 58 [53-65] 125 [113-131] 2.81 [2.31-3.14]	12 11 12 12 12	5.2 [4.7-6.0] 41 [36-56] 58 [42-66] 127 [118-134] 3.35 [2.68-4.06]	0.58 0.20 0.44 0.34 **0.019**

Data are presented as median [IQR] for continuous variables and as n (%) for nominal data. body mass index (BMI), deletion (del), estimated glomerular filtration rate (eGFR), growth hormone (GH), hemoglobin A1c (HbA1c), imprinting center defect (ICD), low-density lipoprotein (LDL), maternal uniparental disomy (mUPD), not available (NA). ^a^ Data presented for n=160 as in two Dutch patients, only urine dipstick was available. ^b^ del vs mUPD. ^c^ not available because of small numbers. ^d^ After adjusting for BMI, p=0.002. ^e^ p=0.013 after adjusting for BMI. ^f^ After adjusting for BMI, p=0.032.

Bold values mean a statistically significant difference.

**Table 3:** Clinical characteristics and laboratory results of nine Dutch adults with PWS and microalbuminuria or elevated UACR.

	Case 1	Case 2	Case 3	Case 4	Case 5	Case 6	Case 7	Case 8	Case 9
CKD stage (35)	G?A2^a^	G?A2^a^	G1A1	G1A2	G1A2	G1A3	G1A2	G1A2	G1A2
**Age** at urine sample, years	29	47	25	36	25	26	27	52	25
**Gender**	Female	Female	Female	Male	Male	Female	Female	Female	Male
**Genotype**	Del, atypical	mUPD	Del type 2	Del, atypical	mUPD	Del type 1	Other	Del type 2	Del, atypical
**BMI** (kg/m^2^)	28.6	31.8	40.8	23.8	28.1	37.4	23.3	38.5	21.8
**Alcohol** usage/week **Smoking**/day	0 0	NA NA	0 0	NA NA	1 0	NA NA	1.5 0	NA 0	0.8 7
**GH treatment**	Never	Never	Only as child	Only as child	Started as child, ongoing	Only as child	Started as child, ongoing	Never	Started as child, ongoing
**Comorbidities** Diabetes Pre-existing hypertension Hypertension treatment Blood pressure systolic/diastolic (mmHg) Hypercholesterolemia Hypothyroidism Hypotthyroidism treatment	No No NA 141/87 No No No	No Yes None 190/95 No Subclinical No	No Yes ATII NA No No No	No No NA NA No No No	IGT Yes CA 156/105 No No No	No No ACE-I 145/106^b^ No Yes Yes	IGT No NA 140/80 No Yes Yes	No Yes ACE-I 137/79 In the past Yes Yes	No No NA 122/66 No No No
**Serology** Serum creatinine (µmol/L) eGFR (mL/min/1.73m^2^) LDL cholesterol (mmol/L)	NA NA NA	NA NA NA	47 132 3.2	67 117 2.1	74 122 2.8	65 113 4.1	56 123 3.5	66 92^c^ 4.0	59 134 2.1
**Urine** Protein Leukocytosis Nitrite Total protein (g/L) UPCR (mg/mmol) Microalbuminuria (mg/L) UACR (mg/mmol)	Neg Pos Pos <0.06 NA 6.0 3.8	Trace Pos Neg 0.15 30.1 61.3 12.3	Trace 2+ Pos 0.08 0.07 20.6 1.9	+ 1+ Neg 0.2 NA 26 NA	Trace Neg NA 0.22 11.3 80.7 4.1	+ Neg Neg 0.77 74.0 570 54.8	Neg 4+ Neg 0.12 43.0 65 23.2	Neg Pos Neg NA NA 37.4 10.7	NA NA NA 0.05 11.0 25 5.8
**Persistent microalbuminuria**	NA	NA	NA	NA	Yes	Yes	No	NA	No

Angiotensin-converting enzyme inhibitor (ACE-I), angiotensin-II receptor antagonist (ATII), body mass index (BMI), calcium antagonist (CA), chronic kidney disease (CKD), deletion (del), estimated glomerular filtration rate (eGFR), growth hormone (GH), impaired glucose tolerance (IGT), low-density lipoprotein (LDL), maternal uniparental disomy (mUPD), not available (NA), urinary albumin-to-creatinine ratio (UACR), urinary protein-to-creatinine ratio (UPCR). ^a^ No recent eGFR available, based on previous laboratory results, CDK stage for both case 1 and 2 would be G1A2. ^b^ 30 minute blood pressure evaluation was performed as hypertension was not previously diagnosed, 24 hour urine sample showed evident proteinuria and patient was treated with an ACE-inhibitor. ^c^ When applying PWS adjusted reference values, eGFR is mildly decreased (<93 mL/min/1.73^2^ in females).

Urine albumin was elevated in 24 of 157 subjects (15%). The UACR was calculated in 75 patients, as urine creatinine was not systematically assessed in all patients, and was abnormal in 19 of 75 patients (25%), of whom 15 had microalbuminuria and four had macroalbuminuria. UPCR was available for 57 patients, of whom ten (18%) had proteinuria and one nephrotic proteinuria (i.e. 460 mg/mmol).

We did not find any association between elevated urine microalbumin or (micro)albuminuria and age, gender, genotype, GH treatment, alcohol consumption or smoking (**Table 2**). Three of 28 patients with normal BMI, three of 37 who were overweight and 22 of 95 with obesity had elevated urine microalbumin or (micro)albuminuria. Patients with elevated urine microalbumin or (micro)albuminuria had significantly higher BMI (38.7 vs 32.4 kg/m^2^, *p*=0.027) and obesity was more prevalent (22 out of 28 (79%) vs 73 of 132 (55%), *p*=0.023). DM2 and hypertension were more prevalent in those with elevated urine microalbumin or (micro)albuminuria than without (57% vs 21%, *p*<0.001 and 39% vs 13%, *p*<0.001 respectively). After adjusting for BMI as possible confounder, these variables remained significantly associated with elevated urine microalbumin or (micro)albuminuria (*p*=0.02 and *p*=0.013 respectively). Laboratory results on glucose, HbA1c, creatinine, eGFR did not differ significantly between the patients with and without elevated urine microalbumin or (micro)albuminuria. However, LDL cholesterol was significantly higher in those with elevated urine microalbumin or (micro)albuminuria than those without (3.35 [2.68-4.06] vs 2.81 [2.31-3.14], *p*=0.019).

Characteristics, comorbidities, and laboratory results of the Dutch patients with elevated urine microalbumin or UACR are shown in **Table 3**. In the Dutch cohort, six out of nine (67%) patients with elevated UACR or UPCR also had leukocyturia and in two subjects, nitrite was also positive. Patients with both leukocyturia and nitrite in the urine sample were advised to have their urine checked for urinary tract infection at the general practitioner. Two patients had transient microalbuminuria or proteinuria (**Table 3**), one of them had leukocytosis at dipstick analysis. Neither had hypertension. Two other Dutch patients had microalbuminuria/proteinuria that persisted after repeating the urine sample. In one case (case 6) a 24-hour urine sample was collected that showed an elevated total protein of 0.56g/24h). A 30-minute blood pressure measurement at home was carried out, which showed systolic and diastolic hypertension. During analysis of hypertension, psychosocial stress turned out to be a strong contributing factor. This patient was referred to our neuropsychologist for psychosocial support and stress management. In addition, an angiotensin-converting enzyme (ACE) inhibitor was prescribed.

#### Leukocyturia in the Dutch cohort

Urine dipstick in the Dutch cohort was positive for leukocytes in 18 of 55 (33%) patients and nitrite in two of 54 (4%). In males, leukocyturia was seen in four of 33 patients (12%, and there was 1+ protein in two, 2+ protein in one and amount unknown in one patient). In females, 14 out of 22 (64%) had leukocyturia with 1+ protein in two, 2+ protein in four, 3+ protein in one, 4+ protein in two and amount unknown in five patients. As in the non-PWS population, leukocyturia was more prevalent in females than in males (*p*<0.001).

#### Glucosuria in the Dutch cohort

Urinary glucose was present in three out of 51 (6%) patients, all of whom had DM2 and were already on a treatment regime. Non-fasting (random) blood glucose levels of these patients ranged from 11.9 to 17.4 mmol/L and HbA1c from 62 to 70 mmol/mol, indicating suboptimal glucose management. None of them had proteinuria.

### Systematic literature review

**Figure 1:**
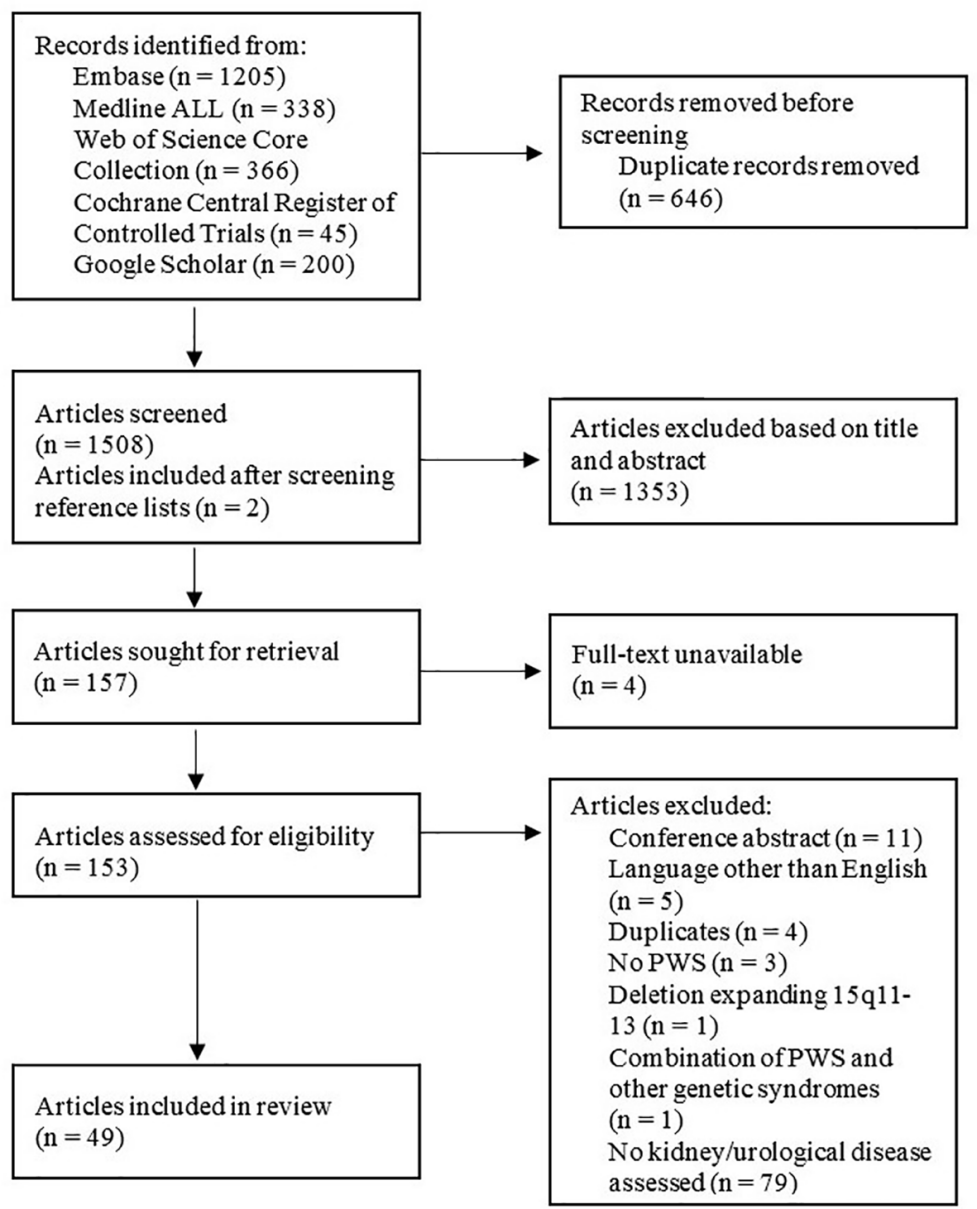
Systematic literature review (63).

Forty-nine publications were included in the literature search (**Figure 1** *).* Multiple case reports have been published on patients with PWS and kidney disease. We found eight case reports describing proteinuria (64–71). There were three case reports in which albuminuria was reported in patients with PWS and DM2 (72–74). One article described a patient with stage 3 CKD (75). Five cases were found describing acute kidney injury (76–80) due to several different causes: pneumoperitoneum with abdominal compartment syndrome (76), secondary rhabdomyolysis caused by hyperpyrexia (77), nephrotoxic medication (78), hyperosmolar hyperglycemic state (79) and respiratory failure due to obesity hypoventilation syndrome (80). We found two case reports describing adults with PWS who needed hemodialysis due to end stage kidney disease, one patient from intrinsic nephrotic disease, the other case from uncontrolled diabetes mellitus (81, 82). Congenital renal or urological abnormalities, such as a horseshoe kidney, bilateral non-communicating paraurethral meatus, and absence of one kidney were also reported (83–85). Multiple UTI were reported in one case report (86) and a congenital disorder (megacystic-microcolon-intestinal hypoperistalsis syndrome) in another (87). Two articles reported on potential underlying genetic components: i) the *NDN* gene which is on the PWS region and ii) *NIPA2*, a gene not imprinted in patients with PWS that influences magnesium transportation, and that possibly reflects an association between kidney homeostasis or disease and PWS (88, 89). Three studies reported on the use of specific medications, such as ACE-inhibitors and (loop)diuretics, that could influence kidney function (90–92). One article mentioned the use of these drugs, without reporting on kidney function (90). In two other articles about these drugs, kidney function was normal (91, 92).

#### Kidney disease

We found 16 studies reporting on kidney disease in patients with PWS (**Table 4**). Sinnema et al. (17) reported a prevalence of urinary tract problems or kidney disorders in 6% (six of 102 adults) with a confirmed diagnosis of PWS. In these patients, blood and urine analysis were performed based on a clinical suspicion of urine tract or kidney problems. Systematic screening of urine or blood samples was not performed in these patients.

**Table 4:** Results of studies reporting on PWS and kidney diseases with more than one patients.

Author (year)	Study design	Method	Baseline characteristics:	Results	Limitations/remarks
Kidney disease					
Sinnema et al. (2011) (17)	Cross sectional study	Semi-structured interview with patient and main caregivers and review of medical files.	N=102 Age: mean 36.2 (range 18-66) years BMI: mean 32.2 (SD ±7.9, range 18.6-51.9) kg/m^2^ Gender: 49M, 53F Genotype: 55 del, 44 mUPD, 3 ICD	Urinary tract/kidney problems were present in 6 (6%) of patients. No association with genotype (del vs mUPD) was found.	No distinction between urinary tract and kidney problems.
Tsuchiya et al. (2011) (48)	Cross-sectional study	Data collected on medical history, patient characteristics, blood samples and urine samples. Proteinuria was defined as UACR of ≥300 mg/gram creatinine and microalbuminuria as UACR of 30-300 mg/gram creatinine. Confirmed by at least two urine samples.	N=65, N=17 with DM Age: median 19 (range 10-53) years BMI: range 27.6-68.2 kg/m^2^ Gender: 43M, 22F Genotype: 52 del, 13 mUPD	Proteinuria was present in 1 out of 17 (6%) and micro-albuminuria in 4 out of 17 (24%) of patients with both PWS and DM. All patients with diabetic nephropathy had a deletion genotype and all but one subject with microalbuminuria were male. Duration of diabetes ranged from 3 to 18 years.	Proteinuria not confirmed by collecting 24-hour urine. Diabetes subtype not specified.
Schmidt et al. (2012) (49)	Retrospective cohort study	Data on patient characteristics, diagnosis laboratory measurements collected from 309 treatment centers in Germany and Austria on patients with DM.	N=23, all with DM Age: mean 16.39 (SD ±3.03) years BMI: mean 37.9 (SD ±11.04) kg/m^2^ Gender: 8M, 15F Genotype: NA	13 out of 23 (56%) were diagnosed with microalbuminuria and three out of 23 (11%) with macroalbuminuria in patients with PWS with DM.	Definition of micro- and macroalbuminuria unknown. Diabetes subtype not available for all patients.
Höybye et al. (2015) (93)	Cross-sectional study	Data collected on medical records, physical examination, blood samples. Comparison between GHt started during childhood and adulthood.	N=10 Age: - childhood group mean 16 (SD ±4) years - adulthood group mean 44 (SD ±4) years BMI: - childhood group mean 32.3 (SD ±10.3) kg/m^2^ - adulthood group 28.9 (SD ±4.6) kg/m^2^ Gender: 10M, 0F Genotype: N=10 methylation positive GHt: N=5 started in childhood, N=5 started as adults, all>5 years treated	One out of 10 patients (10%) was diagnosed with renal insufficiency. Four out of 10 patients (40%) were diagnosed with diabetes mellitus.	No information on cause and severity of renal insufficiency.
Yang et al. (2017) (50)	Retrospective cohort study	Data collected from medical records and screening for DM complication.	N=84, N=29 with DM2 Age: mean 17.4 (SD ±5.1, range 10.3-35.8) years BMI: mean 30.8 (SD ±9.6) kg/m^2^ Gender: 52M, 32F Genotype: 59 del, 25 not specified Ethnicity: Asian	Seven of 29 patients with DM2 (24%) had microvascular complications of whom two (7%) microalbuminuria and one (3%) proteinuria. All three had deletion genotype age between 22.5 – 27.0 years at the onset of microvascular renal complication. Microvascular complications (including albuminuria, retinopathy and peripheral neuropathy) were associated with increased age (r=0.393, *p*=0.047).	Patients with pre-existing chronic kidney disease were excluded from analysis
Koizumi et al. (2018) (94)	Retrospective cohort study	Data collected from medical records on patient characteristics, body composition, laboratory results and CT analysis to assess VAT on t=6 and 12 months after cessation of GHt.	N=7 Age: at end of GHt mean 18.9 (SD ±1.8) years BMI: mean 24.2 (SD ±6.5) kg/m^2^ Gender: 3M, 4F Genotype: all del	One patient (14%) was diagnosed with proteinuria and was taking an ACE- inhibitor before the onset of the study.	No data available on cause, severity or progression of proteinuria/CKD after cessation of GHt.
Van Nieuwpoort et al. (2018) (95)	Cross-sectional cohort study	Data collected on patient characteristics laboratory results including blood and urine samples, bone metabolism and bone mineral density. Data was compared with n=14 healthy siblings.	N=15 Age: median 22.2 (range 19.2-42.9) years BMI: median 27.5 (IQR [16.7]) kg/m^2^ Gender: 4M, 11F Genotype: 14 del, 1 mUPD	The serum creatinine (median, [IQR]) in the whole PWS group was 69.0 [10.0] µmol/L. No significant difference was found in males 71.0 [28.0] µmol/L compared to females 69.0 [10.0] µmol/L, *p*>0.05. Median [IQR] urine creatinine in the total cohort 1.74 [1.47] mmol/2 h, no significant difference was found in males 3.27 [2.86] mmol/2 h compared to females 1.70 [0.69] mmol/2 h.	(micro)albuminuria was not assessed in urine samples.
Manzardo et al. (2019) (96)	Retrospective cohort study	Survey filled in by parents or caregivers.	N=1067 Age: mean 21.0 (SD ±14, range 0-63) years BMI: mean 28.9 (SD ±12, range 3.6-104) kg/m^2^ Gender: 513M, 554F Genotype: 527 del, 325 mUPD, 23 ICD	20 patients (2%) had renal dysfunction of whom 6 out of 38 (17%) had suffered from a thromboembolism vs 14 out of 1013 (1%) with no thromboembolism, *p*<0.0001. Kidney failure increases the risk for thromboembolism (OR 14.9, 95% CI 5.3 – 41.9).	Kidney dysfunction not specified. Severity of kidney failure unknown.
Pemmasani et al. (2021) (47)	Retrospective cohort study	Data collected from the Healthcare Cost and Utilization Project Nationwide Readmissions Database year 2014 on comorbidities of hospitalized patients.	N=480 Age: mean 27 (SD ±19) years Gender: 242M, 238F BMI: NA Genotype: NA	31 patients (7%) were diagnosed with chronic kidney disease. - Ages 0-12 years: <10 out of 132 - Ages 13-25 years: <10 out of 108 - Ages 26-39 years: <10 out of 112 - Ages ≥40 years: 14 out of 128 (11%)	
Cause of death or post mortal analysis					
Cohen et al. (1975) (97)	Case series/retrospective cohort study	Autopsy reports of kidneys of patients with PWS compared to kidney of two age-matched control. Kidneys were both macro- and microscopically analyzed.	N=3 Age: range 3.6– 22 years BMI: NA Gender: 3M Genotype NA CHF: 1	Urinalysis was negative for protein in two PWS patients and not done in one. One patient died of aspiration pneumonia, one of massive pulmonary embolism and the last after infectious complications. Combined weight of the kidneys in all patients were not greater than expected. In the patients with PWS, smooth capsular surfaces, widened cortices and absence of scarring was observed on the kidneys. The mean area of Bowman’s capsule and glomerular tuft were increased in all three PWS patients compared to the controls. Glomerular enlargement was seen, as well as mild dilatation of capillaries and increased cellularity (mainly mesangial origin). No changes consisted with diabetic nephropathy were seen in all patients.	PWS not genetically confirmed.
Nagai et al. (2005) (98)	Retrospective cohort study	Data on cause of death of patients without GHt collected from Japanese patient support societies (group A). Data collected on cause of death from patients with GHt from medical literature (group B).	Group A, no GHt: N=13 Age: range 9 months – 34 years BMI: range 12 – 45.7 kg/m^2^ Gender: 7M, 6F Genotype: 11 del, 1 mUPD, 1 unknown Group B, GHt: N=7 Age: range 0.7-15 years BMI: NA Gender: 7M, 0F Genotype: 3 del, 4 unknown	Cause of death of two patients (15%, aged 28 and 34 years) in group A was for one patient renal and cardiac failure due to DM and the other a pulmonary embolism, renal and cardiac failures. No patient with GHt died from renal failure.	GHt group consisted of only children (age <15 years old).
Butler et al. (2017) (24)	Retrospective database study	Data collected on cause of death from survey filled in by family/caregivers and medical reports of deceased PWS patients.	N=486 Age at death: mean 29.5 (SD ±16, range 2 months-67 years) years BMI (N=132): mean 49.3 (SD ±23, range 14-122) kg/m^2^ Gender: 263M, 217F Genotype: NA	Seven out of 312 (2%, mean age 34.2 (SD ±11 years)) of patients died from renal failure all of whom were >18 years old. Cause of death due to obesity was reported in 22 out of 312 (7%) of patients, all but one patient were >18 years old. Death due to obesity-related factors (CVD, cardiovascular failure, renal failure), appeared in childhood and increased in adolescence and adulthood.	Cause of death available in only 312 out of 486 included patients. Autopsy performed in only 8%, might lead to underestimation of kidney diseases diagnosis.
Pacoricona et al. (2019) (99)	Retrospective observational study	Data collected on cause of death from the French Epidemiological Center for the Medicale Causes of Death Registry and French Reference Center for PWS database from 2004 to 2014 Survey filled in by physician based on medical history and physical examination and a survey filled in by family	N=104 Age at death: median 30 (range 0.1-58) years BMI: NA Gender: 56M, 48F Genotype: 25 del, 9 mUPD, 4 ICD, 66 unknown	One out of 104 (1%) died from sepsis of unknown origin, previously diagnosed with CKD, hypertrophy of the left ventricle with arrhythmia and diabetes. One out of 104 (1%) died suddenly from end-stage renal failure.	Genetic information missing in 63% of patients.

Angiotensin-converting enzyme (ACE), body mass index (BMI), cardiovascular disease (CVD), chronic heart failure (CHF), chronic kidney disease (CKD), computed tomography (CT), confidence interval (CI), deletion (del), diabetes mellitus (DM), dual-energy X-ray absorptiometry (DXA), female (F), glomerular filtration rate (GFR), growth hormone treatment (GHt), imprinting center defect (ICD), interquartile range (IQR), males (M), maternal uniparental disomy (mUPD), not available (NA), Prader-Willi syndrome (PWS), standard deviation (SD), urinary albumin-to-creatinine ratio (UACR), visceral adipose tissue (VAT).

In patients with PWS and diabetes, three studies reported microalbuminuria (7-56%) or proteinuria (3-11%) (48–50). In 480 patients with PWS, Pemmasani et al. (47) reported a prevalence of CKD of 6.5% (31 patients), that increased with age. Renal insufficiency was reported by Höybye et al. (93) in one out of ten patients with PWS who were treated with GHt. Koizumi et al. studied body composition in seven patients with PWS, one of whom was diagnosed with proteinuria and was taking an ACE-inhibitor (94). Van Nieuwpoort et al. reported a median [IQR] serum creatinine in 15 patients of 69.0 [10.0] µmol/L with no significant difference between men and women. According to Manzardo et al. (96) renal dysfunction was more prevalent in patients with PWS who had been diagnosed with thromboembolism than those without (6 out of 34 (17%) vs 14 out of 1013 (1.4%), *p*<0.001).

Multiple studies have investigated causes of death in PWS. Cohen et al. (97) described autopsy results in three children with PWS in whom urinalysis was negative for proteinuria. The kidneys had normal weight but showed glomerular enlargement and dilatation of capillaries as well as increased cellularity compared to controls, suggesting hyperfiltration. Nagai et al. (98) compared 20 patients with PWS with (age range 0.7 to 15 years) and without (never received, age range 9 months to 34 years) GHt. In the group that did not receive GHt, two patients died from renal and cardiac failure, versus none in the GHt group. Two other studies investigating causes of death in PWS both found that 2% died with or because of CKD (24, 99).

#### Urological disorders, diseases and congenital anomalies of kidney and urinary tract

Results of studies reporting urological disorders in PWS are shown in **Table 5**. Torrado et al. (104) reported that five out of 180 (3%) patients with PWS had Congenital Anomalies of Kidney and Urinary Tract (CAKUT), which was significantly higher than in the general population (0.1-0.5%) (*p*<0.05). Malformations found included renal hypoplasia, ureteral duplication, bifid renal pelvis, vesicoureteral reflux, pelvicalyceal dilatation and ureteral valves (104). No association between genotype and reno-ureteral malformations was found (*p*=1.00). Sinnema et al. (101) found CAKUT (bilateral duplication of kidney and ureter system) in one of 12 patients and Pacilli et al. (105) found a horseshoe kidney in one of 33 patients.

**Table 5:** Results of studies reporting on PWS and urological diseases, symptoms or congenital anomalies with more than one patients.

Author (year)	Study design	Method	Baseline characteristics:	Results	Limitations/remarks
Von Gontard et al. (2010) (100)	Cohort study	Questionnaire filled in by parents/caregivers	N=118 Age: mean 20.5 (SD ±11.5, range 5-45) years BMI: NA Gender: NA Genotype: 32 del, 27 mUPD, 3 ICD, 56 other/unknown	16 out of 118 patients (14%) had nocturnal enuresis of whom nine (56%) between the age of 5-12. Only four adults (7%) experienced nocturnal enuresis. A total of five out of 118 patients (4%) had additional daytime urine incontinence. The mean age in this group was 21.4 (SD ±14.8) years. Urgency symptoms were seen in nine out of 16 patients (60%) with nocturnal enuresis. Five out of 16 patients (32%) had a history of UTIs and only 2 (13%) a history of UTIs with fever.	
Sinnema et al. (2012) (101)	Retrospective cohort study	Semi-structured interviews with patient and main caregivers, questionnaire filled in by parents/caregivers.	N=12 Age: mean 57.8 (SD ±6.2, range 50-66) years BMI: 31.5 (SD ±5.,0 range 23.4-37.1) kg/m^2^ Gender: 5M, 7F Genotype: 4 del, 8 mUPD	Kidney malformations were present in only one out of 12 (8%) patients, which was a bilateral duplication of the kidney and ureter system.	Study population overlapping with Sinnema et al., 2011 (17).
Equit et al. (2013) (102)	Cross-sectional study	Questionnaires filled in by parents/caregivers of self-help groups. Comparison with patients with fragile X-syndrome.	N=191 Age: mean 20.0 (SD ±10.5) years BMI: NA Gender: 104M, 87F Genotype: NA	56 out of 191 patients (29%) had at least one elimination disorder. - 42 out of 191 (22%) suffered from nocturnal enuresis and 23 patients (12%) from daytime urinary incontinence. Daytime urinary incontinence was more prevalent in patients with fragile X-syndrome than PWS (*p*<0001). The prevalence of elimination (NE, DIU or FI) disorders decreased with increasing age. - At age 4-12 had 18 out of 54 (33%) at least one elimination disorder, at age 13-17, this was 16 out of 47 (34%), at age 18-30 15 out of 60 (25%) and 30 years or older six out of 29 (21%) Urgency symptoms were present in 49 out of 191 (26%) of patients with PWS. 42 out of 191 (22%) were previously diagnosed with UTIs. More patients with PWS had have previous UTI’s compared to fragile X syndrome (42 out of 191 (22%) versus 18 out of 166 (11%), *p*=0.005).	Return rate of 48.9% for patients with PWS might have led to selection bias.
Meinhardt et al. (2013) (103)	Retrospective observational study	Data collected on anthropometric measurements, laboratory results and DXA scans at baseline, after one year and at last observation.	N=41 Age: mean 3.8 (SD ±3.0, range 0.4-12.2), years BMI: mean 0.6 (SDS ±1.9) Gender: 22M, 19F Genotype: NA GHt: all, none treated before study	One patient (2%) suffered from a severe UTI and convulsion during GHt after which treatment was stopped after 2.4 years and patient fully recovered.	
Torrado et al. (2013) (104)	Retrospective cohort study	Data collected from medial reports of PWS compared. Prevalences of birth defects in PWS compared to general population by collecting data from multiple population registries.	N=180 Age at diagnosis 1.2 (range 0.01-17.25) years BMI: NA Gender: 93M, 87F Genotype: 109 del, 68 mUPD, 3 ICD	Five out of 180 patients (3%, one female and four males) had congenital reno-ureteral malformation; left renal hypoplasia, bilateral ureteral duplication, left bifid renal pelvis, vesicoureteral reflux, left pelvicalyceal dilatation and bilateral vesicoureteral reflux caused by ureteral valves. The prevalence of reno-ureteral malformations was significantly higher than in the general population in national and international registries (*p*<0.05). No association between genotype and congenital malformations was found (three del vs two non del, *p*=1.00).	
Pacilli et al. (2018) (105)	Cohort study	Data collected from patient records from the Victorian PWS Register.	N=33 Age: range 6 months – 17 years BMI: NA Gender: all male Genotype: NA	One out of 33 (3%) patients that underwent orchidopexy was diagnosed with a horseshoe kidney.	
Chao et al. (2021) (34)	Retrospective observational study	Data collection on patient characteristics and presence of LUTS. Assessments of LUTD by uroflowmetry, postvoid residual urine (PVR) by abdominal ultrasound. LUTD defined as abnormal uroflow, low peak flow rate (Q_max_) or elevated PVR. Videourodynamic studies were performed in some cases.	N=37 Age: mean 17.7 (SD ±7.8, range 5-34) years BMI: mean 28.5 (SD ±11.2) kg/m^2^ Gender: 15M, 22F Genotype: 16 del, 3 mUPD, 18 unknown	Ten out of 37 patients (27%) had LUTS. The urodynamic tests were abnormal in 17 out of 34 (50%) of patients. Abnormal uroflowmetry pattern (nonbell shaped) was significantly more prevalent in those with LUTS than without (6 out of 8 (75%) versus 6 out of 20 (23%) respectively, *p*=0.0049). In patients with LUTS (n=10), 4 (40%) had daytime UI, 2 (20%) both daytime and nighttime UI, one (10%) had nocturnal enuresis and three (30%) LUTS without UI. In 10 out of 20 patients (50%) PVR was increased for their age. Q_max_, voided volume and bladder capacity were significantly higher in those without LUTS (24.0 (9.0) vs 14.4 (13.5) ml/s, *p*=0.0046; 240.6 (124.0) vs 126.4 (91.8) ml, *p*=0.0142 and 247.7 (123.2) vs 143.5 (121.1) ml, *p*=0.0151 respectively). In three LUTS patients in whom videourodynamic studies were performed, all showed detrusor sphincter dyssynergia.	Low compliance to PVR (54%).

Body mass index (BMI), congenital anomalies of the kidney and urinary tract (CAKUT), daytime urinary incontinence (DIU), deletion (del), dual-energy X-ray absorptiometry (DXA), fecal incontinence (FI), female (F), growth hormone treatment (GHt), imprinting center defect (ICD), lower urinary tract dysfunction (LUTD), lower urinary tract symptoms (LUTS), male (M), maternal uniparental disomy (mUPD), nocturnal enuresis (NE), not available (NA), Prader-Willi syndrome (PWS), postvoid residual urine (PVR), standard deviation (SD), urinary incontinence (UI), urinary tract infection (UTI).

Nocturnal enuresis was described in 14-22% (100, 102). Two studies found a decrease in nocturnal enuresis with increasing age (100, 102). Daytime urinary incontinence was present in 3-12% (100, 102). Urgency symptoms, possibly related to overactive bladder, were reported in 26-60% of whom 22-32% had a history of UTI’s (100, 102).

Chao et al. (34) reported that urodynamic tests were abnormal in 17 out of 34 patients with PWS, with abnormal flowmetry in 12 out of 34 and elevated residual bladder volume in 10 out of 20. In patients with lower urinary tract symptoms (LUTS), peak flow rate, voided volume, and bladder capacity were significantly decreased compared to patients without LUTS.

## Discussion

Our study in 162 adults with PWS showed that one in every five (young) adults had early signs of microvascular disease, measured by elevated urine microalbumin or (micro)albuminuria, while eGFR was normal. However, as muscle mass is low in PWS (6), creatinine levels and eGFR may be falsely ‘normal’. Our findings suggest that pre-symptomatic kidney injury may be missed when only blood measurements are performed. Therefore, in this patient group, urinalysis is essential for timely screening of microvascular (kidney) disease. We propose an algorithm for the detection and management of microvascular disease in adults with PWS.

Twenty-eight out of 160 (18%) patients had elevated urine albumin, (micro) albuminuria or proteinuria; eleven of whom were diagnosed with hypertension and sixteen with DM2. No age difference was found between patients with normal and elevated UACR.

In our literature review, we found an overall prevalence of microalbuminuria of 7-56% and macroalbuminuria of 3-11% in patients with PWS and diabetes (48–50). We did not find any studies on microalbuminuria or proteinuria in patients with PWS without DM2. Our study is the first to analyze the presence of (micro)albuminuria in patients with PWS with and without DM2. In the general population, around 25% of patients with longstanding (>10 years) DM2 have microalbuminuria and 5-20% proteinuria (51). Before antihypertensive treatment, patients with essential hypertension (in the general population) developed proteinuria in 35-65% and renal insufficiency in 33%, depending on severity of hypertension, DM2, medication, and age (106). In our cohort, 16 of 44 (36%) of patients with DM2 had (micro)albuminuria. Notably, the prevalence of albuminuria in our PWS patients *without* DM2 was similar to that in non-PWS patients *with* long-term DM2. The albuminuria in our PWS patients suggests microvascular damage is already present at a young age (23-33 years).

In this study, as in the normal population, albuminuria was associated with hypertension, DM2 and obesity. Obesity is associated with chronic kidney disease and glomerulosclerosis. A possible mechanism is hyperfiltration, which leads to albuminuria, regardless of the presence or absence of diabetes (107–110). Furthermore, obesity has been associated with increased progression of CKD (111).

In the general population, microalbuminuria is an indicator for microvascular injury, and a risk factor for both CKD and CVD (40–42, 53, 54). Although none of our patients was formally diagnosed with CVD, the presence of microalbuminuria might indicate latent CVD. Young adults with PWS often have cardiovascular risk factors including obesity (60%), DM2 (27%), hypertension (18%), smoking (8%) and hypercholesterolemia (14%) or dyslipidemia (10%). Timely and adequate follow-up of microalbuminuria and treatment of these risk factors might decrease cardiovascular and renal comorbidities and complications.

### Clinical recommendations

#### Initial visit

During the initial visit, the physician should ask about previous UTIs and use of cigarettes and/or alcohol. In case of multiple UTIs, one should consider a kidney ultrasound to detect malformations, assess the post-void residual urine volume and referral to a urologist. In case of substance use, we advise to encourage cessation of smoking or alcohol usage. Furthermore, weight and blood pressure should be measured yearly. In case of hypertension, one should consider analysis to secondary causes such as stress, left ventricle hypertrophy or renal causes and manage both obesity and hypertension according to general guidelines.

#### Monitoring of eGFR

**Figure 2:**
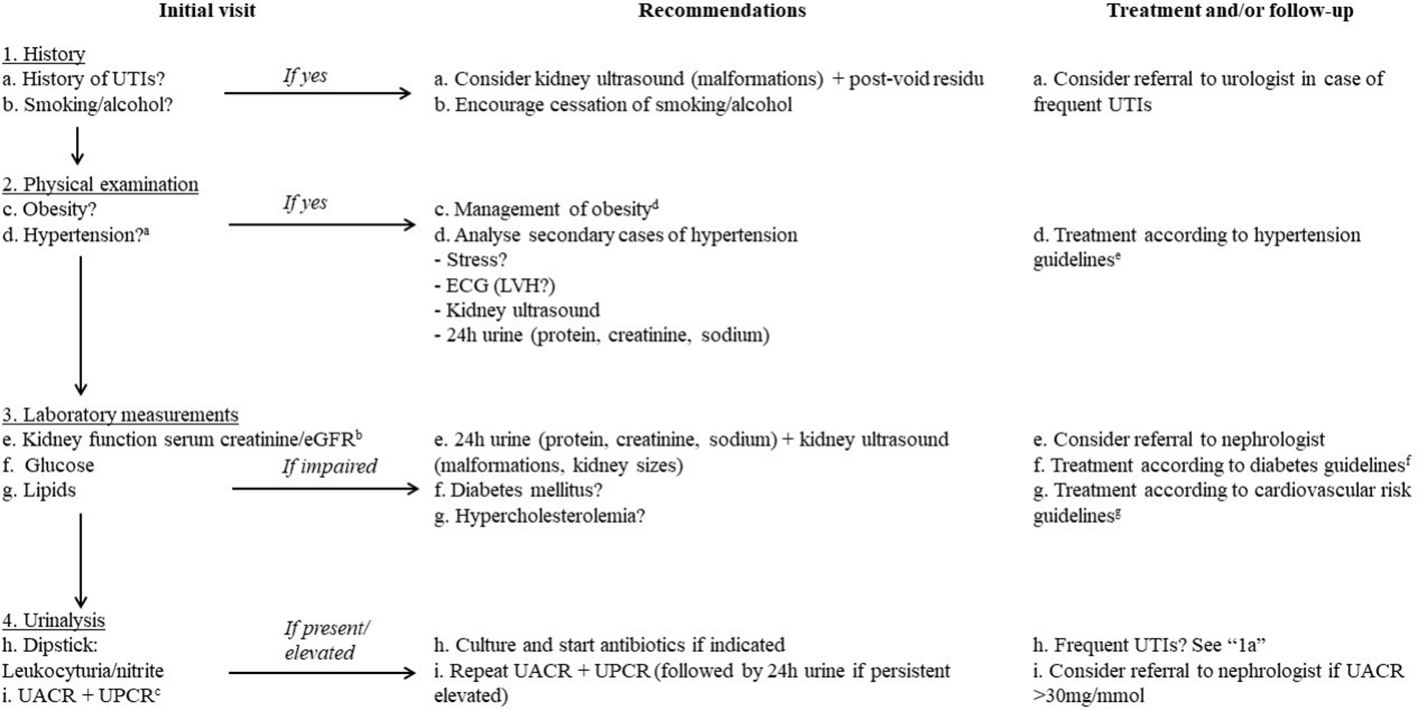
Recommendations for screening and the management for (micro)albuminuria and its risk factors in adults with PWS. angiotensin-converting enzyme inhibitor (ACE-I), angiotensin-II receptor antagonist (ATII) electrocardiogram (ECG), estimated glomerular filtration rate (eGFR), Left ventricular hypertrophy (LVH), minutes (min), urinary tract infection (UTI), urinary albumin-to-creatinine ratio (UACR), urinary protein-to-creatinine ratio (UPCR). a. Diagnosed by repeated measurements or 30-minute measurement. b. preferably in a first morning urine sample. c. Consider using adjusted reference values for adults with PWS (e.g. >98 ml/min/1.73m2 in males and >93 ml/min/1.73m2 in females) (20). d. Obesity in adults with PWS should be aggressively management with for example a hypocaloric diet, structured regular exercise and restriction of access to food. e. For example the 2018 ESC/ESH guidelines for the management of arterial hypertension (116). f. For example the 2019 ESC guidelines on diabetes (117). g. For example the ESC 2021 guideline (118) on cardiovascular disease prevention.

In our study, we showed that median creatinine levels were at the lower limit of the reference interval of the general (non-PWS) population in both male and female adults with PWS. This finding could be related to low muscle mass, which is decreased by 25 to 37% in patients with PWS (6), resulting in an overestimation of kidney function when measured by eGFR. The eGFR (calculated using the CKD-EPI) might therefore not be the optimal test to estimate kidney function in adults with PWS. We previously proposed alternative reference values of eGFR for adults with PWS (>98 ml/min/1.73m^2^ in males and >93 ml/min/1.73m^2^ in females) (20) to avoid underdiagnosis of CKD. Another method of assessing kidney injury is by measuring the excretion of LMW proteins, such as cystatine-C, retinol binding protein and alpha1-microglobulin. LMW proteins are filtered and reabsorbed by the kidneys. In kidney injury and (diabetic) proteinuria, an increased excretion of these proteins might be found in urine samples (44–46). Therefore, a more accurate methods of assessing kidney function in patients with PWS might include cystatin-C clearance adjusted for BMI (112, 113) or 24-hour urine creatinine measurement. However, multiple factors such as thyroid dysfunction and corticosteroid use might influence cystatin-C levels (114, 115). We therefore recommend yearly evaluation of creatinine and eGFR according to PWS-specific cut-off values and yearly urinalysis in all adults with PWS (see **Figure 2**) (20).

#### Laboratory measurements

We advise yearly evaluation of kidney function, glucose levels and lipids. If an impaired renal function is detected, additional investigation using a 24-hour urine and kidney ultrasound should be performed to identify the cause. In addition, a nephrologist could be consulted. A new diagnosis of diabetes or hypercholesterolemia should be managed according to non-PWS guidelines.

#### Detection of UTIs

Urgency symptoms (sudden need to pass urine with or without urine incontinence) and UTIs are common in adults with PWS, according to our literature review and previous study in our cohort (26-60% and 22-32%, respectively) (100, 102, 119). UTIs might be prevalent in adults with PWS related to obesity (120). Health care providers should be aware of the increased pain threshold and inability to mount a fever (due to disturbed temperature regulation) in patients with PWS (8, 32). This combination of features might lead to an atypical presentation of UTI (e.g. only changes in behavior), causing underdiagnosis of UTIs in this vulnerable patient population. If leukocytes and/or nitrite are positive, a urine culture should be performed and the patient should be treated with antibiotics if indicated.

#### Urinalysis for the detection of micro- or macroalbuminuria

We recommend performing urinalysis (midstream urinalysis using urine dipstick and measurement of UACR and UPCR) yearly in all adults with PWS. First morning urine samples are preferred over random urine samples to decrease the influence of orthostatic proteinuria (35). In those diagnosed with hypertension and DM2, we advise stringent control of blood pressure and glucose levels. In addition, we recommend performing urinalysis yearly, as per guidelines for DM2 and hypertension in the general population (116, 117).

In case of abnormalities, a second sample urinalysis should be performed. When microalbuminuria or proteinuria persists, 24-hour urine should be collected to calculate the 24 hour creatinine- and protein excretion. However, in people with of intellectual disability, collecting 24 hour urine might be challenging. In those with normal eGFR but elevated UACR or UPCR without a UTI, an renal ultrasound should be performed.

When proteinuria is confirmed, patients should be screened for underlying diseases and any risk factors should be treated. Cardiovascular status should be optimized and cardiovascular risk should be assessed. Referral to (or consultation with) a nephrologist should be considered. Those with progressive proteinuria should be referred to a nephrologist. In adults with PWS with albuminuria and hypertension, an ACE inhibitor or angiotensin-II receptor antagonist should be the first line of treatment due to their renoprotective effects (121). If obesity is present, a weight reducing regime should be started.

### Strengths and limitations

As all studies, our study has strengths and limitations. This study is, to our knowledge, the first cohort study to systematically assess kidney function and urinalysis in adults with PWS. However, there are several limitations. Blood and urine samples were not always collected on the same day as the blood samples [and in eight Dutch and all French patients, blood results were not included in the statistical analysis due to the prolonged time interval between blood and urine samples (>12 months)]. As random spot urine samples were collected instead of first morning urine samples, the high prevalence of (micro)albuminuria might partly be caused by sample contamination, orthostatic proteinuria or physical exertion. Furthermore, microalbuminuria was not confirmed in a second sample as repeated samples were only available in four patients. In addition, as muscle mass might be low in patients with PWS, leading to a low serum creatinine, defining microalbuminuria by the UACR or UPCR might have led to an overestimation (6). Smoking and alcohol usage were not assessed yearly in some patients, and data could therefore have been out of date. Patients with leukocyturia or nitrites were first referred to their general practitioner for urine culture and UTI treatment if positive for infection, after which urinalysis was repeated. Therefore, the results of urine cultures were not available at our center. Urinalysis was not available in the entire cohort, which might have led to selection bias and a 24-hour urinalysis was only available in one patient. Additionally, patients were not routinely screened for CVD (e.g. by an ECG or cardiac ultrasound). Furthermore, this cohort consists of relatively young adults without cardiovascular events, though risk factors for CVD are present, which justifies urinalysis even in young patients with PWS. Prospective studies in a larger cohort are needed to overcome these limitations.

## Conclusion

Upon screening, one in every five adults with PWS had elevated urine albumin or (micro)albuminuria which was already present at young age. All had normal eGFR according to non-PWS reference intervals. However, as muscle mass is low in PWS, normal creatinine levels and eGFR may overestimate kidney function in people with this syndrome. As kidney function may be overestimated when only measured by serum creatinine, renal problems may be missed when urinalysis is omitted. Routine screening for microalbuminuria may allow early intervention to avoid CVD and CKD. Health care providers should be aware of the increased risk for CKD and CVD in adults with PWS and should optimize treatment to reduce these risk factors. To prevent long-term complications and impaired quality of life, we provide an algorithm for the screening and management of micro-albuminuria and proteinuria in adults with PWS (**Figure 2**).

## Data availability statement

The raw data supporting the conclusions of this article will be made available by the authors on request, without undue reservation.

## Ethics statement

The studies involving human participants were reviewed and approved by Medical Ethics Commission of the Erasmus Medical Center, Rotterdam, the Netherlands. The patients/participants provided their written informed consent to participate in this study. Written informed consent was obtained from the individual(s) for the publication of any potentially identifiable images or data included in this article.

## Author contributions

Conceptualization, KP, FH and LG. Methodology, KP and LG. Formal analysis, DA. Investigation, DA, NN. Resources, LG. Data curation, DA, NN, GG, CP. Writing—original draft preparation, DA. Writing—review and editing, all authors. Visualization: DA. Supervision, KP, LG and AL. Project administration, DA, KP and LG. All authors contributed to the article and approved the submitted version.
