# Corrigendum: Kidney disease in adults with Prader-Willi syndrome: international cohort study and systematic literature review

**DOI:** 10.3389/fendo.2024.1357219

**Published:** 2024-01-22

**Authors:** Denise H. van Abswoude, Karlijn Pellikaan, Naomi Nguyen, Anna G. W. Rosenberg, Kirsten Davidse, Franciska M. E. Hoekstra, Ilse M. Rood, Christine Poitou, Graziano Grugni, Charlotte Høybye, Tania P. Markovic, Assumpta Caixàs, Antonino Crinò, Sjoerd A. A. van den Berg, Aart J. van der Lely, Laura C. G. de Graaff

**Affiliations:** ^1^ Department of Internal Medicine, Division of Endocrinology, Erasmus Medical Center, University Medical Center Rotterdam, Rotterdam, Netherlands; ^2^ Center for Adults with Rare Genetic Syndromes, Department of Internal Medicine, Division of Endocrinology, Erasmus Medical Center, University Medical Center Rotterdam, Rotterdam, Netherlands; ^3^ Dutch Center of Reference for Prader–Willi Syndrome, Rotterdam, Netherlands; ^4^ Academic Center for Growth Disorders, Erasmus Medical Center, University Medical Center Rotterdam, Rotterdam, Netherlands; ^5^ Internal Medicine, Division of Nephrology, Reinier de Graaf Gasthuis, Delft, Netherlands; ^6^ Department of Nephrology, Radboud University Medical Center, Radboud Institute for Health Sciences, Nijmegen, Netherlands; ^7^ Assistance Publique-Hôpitaux de Paris, Rare Diseases Center of Reference ‘Prader-Willi Syndrome and Obesity with Eating Disorders’ (PRADORT), Nutrition Department, Institute of Cardiometabolism and Nutrition (ICAN), Pitié-Salpêtrière Hospital, Sorbonne Université, National Institute of Health and Medical Research (INSERM), Nutriomics, Paris, France; ^8^ International Network for Research, Management & Education on adults with Prader-Willi Syndrome (INfoRMEd-PWS); ^9^ European Reference Network on Rare Endocrine Conditions (ENDO-ERN); ^10^ Division of Auxology, Istituto Auxologico Italiano, Istituto di Ricovero e Cura a Carattere Scientifico (IRCCS), Piancavallo, Italy; ^11^ Department of Molecular Medicine and Surgery, Karolinska Institute and Karolinska University Hospital, Stockholm, Sweden; ^12^ Department of Endocrinology, Karolinska Institute and Karolinska University Hospital, Stockholm, Sweden; ^13^ Metabolism & Obesity Service, Royal Prince Alfred Hospital, Camperdown, NSW, Australia; ^14^ Charles Perkins Center and Sydney Medical School, University of Sydney, Sydney, NSW, Australia; ^15^ Department of Endocrinology and Nutrition, Parc Tauli Hospital Universitari, Institut d’Investigació i Innovació Parc Taulí (I3PT) Instituto de Salud Carlos III (CERCA-ISCIII), Sabadell, Spain; ^16^ Department of Medicine, Universitat Autònoma de Barcelona, Sabadell, Spain; ^17^ Reference Center for Prader-Willi syndrome, Bambino Gesù Hospital, Research Institute, Palidoro, Italy; ^18^ Center for Rare Diseases and Congenital Defects, Fondazione Policlinico Universitario A. Gemelli, Istituto di Ricovero e Cura a Carattere Scientifico (IRCCS), Rome, Italy; ^19^ Department of Clinical Chemistry, Erasmus Medical Center (MC), University Medical Center Rotterdam, Rotterdam, Netherlands

**Keywords:** Prader-Willi Syndrome, kidney function tests, proteinuria, urine tract infections, cardiovascular disease, kidney disease

In the published article, there was an error in **Table 4** as published. The results of the article by Van Nieuwpoort et al. (2018) were displayed as “Median [IQR] urine creatinine in the total cohort 1.74 [1.47] mmol/24 h, no significant difference was found in males 3.27 [2.86] mmol/24 h compared to females 1.70 [0.69] mmol/24 h.” The correct statement is “Median [IQR] urine creatinine in the total cohort 1.74 [1.47] mmol/2 h, no significant difference was found in males 3.27 [2.86] mmol/2 h compared to females 1.70 [0.69] mmol/2 h”. The corrected **Table 4** and its caption appear below.

**Table 4:** Results of studies reporting on PWS and kidney diseases with more than one patient.

Author (year)	Study design	Method	Baseline characteristics:	Results	Limitations/remarks
Kidney disease					
Sinnema et al. (2011)^(17)^	Cross sectional study	Semi-structured interview with patient and main caregivers and review of medical files.	N=102 Age: mean 36.2 (range 18-66) years BMI: mean 32.2 (SD ±7.9, range 18.6-51.9) kg/m^2^ Gender: 49M, 53F Genotype: 55 del, 44 mUPD, 3 ICD	Urinary tract/kidney problems were present in 6 (6%) of patients. No association with genotype (del vs mUPD) was found.	No distinction between urinary tract and kidney problems.
Tsuchiya et al. (2011)^(48)^	Cross-sectional study	Data collected on medical history, patient characteristics, blood samples and urine samples. Proteinuria was defined as UACR of ≥300 mg/gram creatinine and microalbuminuria as UACR of 30-300 mg/gram creatinine. Confirmed by at least two urine samples.	N=65, N=17 with DM Age: median 19 (range 10-53) years BMI: range 27.6-68.2 kg/m^2^ Gender: 43M, 22F Genotype: 52 del, 13 mUPD	Proteinuria was present in 1 out of 17 (6%) and micro-albuminuria in 4 out of 17 (24%) of patients with both PWS and DM. All patients with diabetic nephropathy had a deletion genotype and all but one subject with microalbuminuria were male. Duration of diabetes ranged from 3 to 18 years.	Proteinuria not confirmed by collecting 24-hour urine. Diabetes subtype not specified.
Schmidt et al. (2012)^(49)^	Retrospective cohort study	Data on patient characteristics, diagnosis laboratory measurements collected from 309 treatment centers in Germany and Austria on patients with DM.	N=23, all with DM Age: mean 16.39 (SD ±3.03) years BMI: mean 37.9 (SD ±11.04) kg/m^2^ Gender: 8M, 15F Genotype: NA	13 out of 23 (56%) were diagnosed with microalbuminuria and three out of 23 (11%) with macroalbuminuria in patients with PWS with DM.	Definition of micro- and macroalbuminuria unknown. Diabetes subtype not available for all patients.
Höybye et al. (2015)^(93)^	Cross-sectional study	Data collected on medical records, physical examination, blood samples. Comparison between GHt started during childhood and adulthood.	N=10 Age: - childhood group mean 16 (SD ±4) years - adulthood group mean 44 (SD ±4) years BMI: - childhood group mean 32.3 (SD ±10.3) kg/m^2^ - adulthood group 28.9 (SD ±4.6) kg/m^2^ Gender: 10M, 0F Genotype: N=10 methylation positive GHt: N=5 started in childhood, N=5 started as adults, all>5 years treated	One out of 10 patients (10%) was diagnosed with renal insufficiency. Four out of 10 patients (40%) were diagnosed with diabetes mellitus.	No information on cause and severity of renal insufficiency.
Yang et al. (2017)^(50)^	Retrospective cohort study	Data collected from medical records and screening for DM complication.	N=84, N=29 with DM2 Age: mean 17.4 (SD ±5.1, range 10.3-35.8) years BMI: mean 30.8 (SD ±9.6) kg/m^2^ Gender: 52M, 32F Genotype: 59 del, 25 not specified Ethnicity: Asian	Seven of 29 patients with DM2 (24%) had microvascular complications of whom two (7%) microalbuminuria and one (3%) proteinuria. All three had deletion genotype age between 22.5 – 27.0 years at the onset of microvascular renal complication. Microvascular complications (including albuminuria, retinopathy and peripheral neuropathy) were associated with increased age (r=0.393, *p*=0.047).	Patients with pre-existing chronic kidney disease were excluded from analysis
Koizumi et al. (2018)^(94)^	Retrospective cohort study	Data collected from medical records on patient characteristics, body composition, laboratory results and CT analysis to assess VAT on t=6 and 12 months after cessation of GHt.	N=7 Age: at end of GHt mean 18.9 (SD ±1.8) years BMI: mean 24.2 (SD ±6.5) kg/m^2^ Gender: 3M, 4F Genotype: all del	One patient (14%) was diagnosed with proteinuria and was taking an ACE- inhibitor before the onset of the study.	No data available on cause, severity or progression of proteinuria/CKD after cessation of GHt.
Van Nieuwpoort et al. (2018)^(95)^	Cross-sectional cohort study	Data collected on patient characteristics laboratory results including blood and urine samples, bone metabolism and bone mineral density. Data was compared with n=14 healthy siblings.	N=15 Age: median 22.2 (range 19.2-42.9) years BMI: median 27.5 (IQR [16.7]) kg/m^2^ Gender: 4M, 11F Genotype: 14 del, 1 mUPD	The serum creatinine (median, [IQR]) in the whole PWS group was 69.0 [10.0] µmol/L. No significant difference was found in males 71.0 [28.0] µmol/L compared to females 69.0 [10.0] µmol/L, *p*>0.05. Median [IQR] urine creatinine in the total cohort 1.74 [1.47] mmol/2 h, no significant difference was found in males 3.27 [2.86] mmol/2 h compared to females 1.70 [0.69] mmol/2 h.	(micro)albuminuria was not assessed in urine samples.
Manzardo et al. (2019)^(96)^	Retrospective cohort study	Survey filled in by parents or caregivers.	N=1067 Age: mean 21.0 (SD ±14, range 0-63) years BMI: mean 28.9 (SD ±12, range 3.6-104) kg/m^2^ Gender: 513M, 554F Genotype: 527 del, 325 mUPD, 23 ICD	20 patients (2%) had renal dysfunction of whom 6 out of 38 (17%) had suffered from a thromboembolism vs 14 out of 1013 (1%) with no thromboembolism, *p*<0.0001. Kidney failure increases the risk for thromboembolism (OR 14.9, 95% CI 5.3 – 41.9).	Kidney dysfunction not specified. Severity of kidney failure unknown.
Pemmasani et al. (2021)^(47)^	Retrospective cohort study	Data collected from the Healthcare Cost and Utilization Project Nationwide Readmissions Database year 2014 on comorbidities of hospitalized patients.	N=480 Age: mean 27 (SD ±19) years Gender: 242M, 238F BMI: NA Genotype: NA	31 patients (7%) were diagnosed with chronic kidney disease. - Ages 0-12 years: <10 out of 132 - Ages 13-25 years: <10 out of 108 - Ages 26-39 years: <10 out of 112 - Ages ≥40 years: 14 out of 128 (11%)	
Cause of death or post mortal analysis					
Cohen et al. (1975)^(97)^	Case series/retrospective cohort study	Autopsy reports of kidneys of patients with PWS compared to kidney of two age-matched control. Kidneys were both macro- and microscopically analyzed.	N=3 Age: range 3.6– 22 years BMI: NA Gender: 3M Genotype NA CHF: 1	Urinalysis was negative for protein in two PWS patients and not done in one. One patient died of aspiration pneumonia, one of massive pulmonary embolism and the last after infectious complications. Combined weight of the kidneys in all patients were not greater than expected. In the patients with PWS, smooth capsular surfaces, widened cortices and absence of scarring was observed on the kidneys. The mean area of Bowman’s capsule and glomerular tuft were increased in all three PWS patients compared to the controls. Glomerular enlargement was seen, as well as mild dilatation of capillaries and increased cellularity (mainly mesangial origin). No changes consisted with diabetic nephropathy were seen in all patients.	PWS not genetically confirmed.
Nagai et al. (2005)^(98)^	Retrospective cohort study	Data on cause of death of patients without GHt collected from Japanese patient support societies (group A). Data collected on cause of death from patients with GHt from medical literature (group B).	Group A, no GHt: N=13 Age: range 9 months – 34 years BMI: range 12 – 45.7 kg/m^2^ Gender: 7M, 6F Genotype: 11 del, 1 mUPD, 1 unknown Group B, GHt: N=7 Age: range 0.7-15 years BMI: NA Gender: 7M, 0F Genotype: 3 del, 4 unknown	Cause of death of two patients (15%, aged 28 and 34 years) in group A was for one patient renal and cardiac failure due to DM and the other a pulmonary embolism, renal and cardiac failures. No patient with GHt died from renal failure.	GHt group consisted of only children (age <15 years old).
Butler et al. (2017)^(24)^	Retrospective database study	Data collected on cause of death from survey filled in by family/caregivers and medical reports of deceased PWS patients.	N=486 Age at death: mean 29.5 (SD ±16, range 2 months-67 years) years BMI (N=132): mean 49.3 (SD ±23, range 14-122) kg/m^2^ Gender: 263M, 217F Genotype: NA	Seven out of 312 (2%, mean age 34.2 (SD ±11 years)) of patients died from renal failure all of whom were >18 years old. Cause of death due to obesity was reported in 22 out of 312 (7%) of patients, all but one patient were >18 years old. Death due to obesity-related factors (CVD, cardiovascular failure, renal failure), appeared in childhood and increased in adolescence and adulthood.	Cause of death available in only 312 out of 486 included patients. Autopsy performed in only 8%, might lead to underestimation of kidney diseases diagnosis.
Pacoricona et al. (2019)^(99)^	Retrospective observational study	Data collected on cause of death from the French Epidemiological Center for the Medicale Causes of Death Registry and French Reference Center for PWS database from 2004 to 2014 Survey filled in by physician based on medical history and physical examination and a survey filled in by family	N=104 Age at death: median 30 (range 0.1-58) years BMI: NA Gender: 56M, 48F Genotype: 25 del, 9 mUPD, 4 ICD, 66 unknown	One out of 104 (1%) died from sepsis of unknown origin, previously diagnosed with CKD, hypertrophy of the left ventricle with arrhythmia and diabetes. One out of 104 (1%) died suddenly from end-stage renal failure.	Genetic information missing in 63% of patients.

ACE, angiotensin-converting enzyme; BMI, body mass index; CVD, cardiovascular disease; CHF, chronic heart failure; CKD, chronic kidney disease; CT, computed tomography; CI, confidence interval; del, deletion; DM, diabetes mellitus; DXA, dual-energy X-ray absorptiometry; F, female; GFR, glomerular filtration rate; GHt, growth hormone treatment; ICD, imprinting center defect; IQR, interquartile range; M, males; mUPD, maternal uniparental disomy; NA, not available; PWS, Prader-Willi syndrome; SD, standard deviation; UACR, urinary albumin-to-creatinine ratio; VAT, visceral adipose tissue.

The authors apologize for this error and state that this does not change the scientific conclusions of the article in any way. The original article has been updated.

