# Supplementary material for: Kidney disease in adults with Prader-Willi syndrome: international cohort study and systematic literature review
Source: Front Endocrinol (Lausanne). 2023 Jul 21;14:1168648. doi: 10.3389/fendo.2023.1168648 (PMC10402738; doi:10.3389/fendo.2023.1168648)
Supplement: Supplementary file 1 [file Table_1.docx]

Supplementary Material

**Kidney disease in adults with Prader-Willi syndrome: international cohort study and systematic literature review**

Denise H. van Abswoude, MD, Karlijn Pellikaan, BSc, Naomi Nguyen, BSc, Anna G.W. Rosenberg, BSc, Kirsten Davidse, MSc, Franciska M.E. Hoekstra, MD, PhD, Ilse M. Rood, MD, PhD, Christine Poitou, MD, PhD, Graziano Grugni, MD, PhD, Charlotte Høybye, MD, PhD, Tania P. Markovic, MD, PhD, Assumpta Caixàs MD, PhD, Antonino Crinò, MD, PhD, Sjoerd A.A. van den Berg, MSc, PhD, Aart J. van der Lely, MD, PhD, Laura C.G. de Graaff, MD, PhD^*^

**Correspondence:**

Corresponding Author:

Laura de Graaff, MD, PhD

E-mail l.degraaff@erasmusmc.nl

**Table S1. Search strategy**

| **Embase**  ('Prader Willi syndrome'/exp OR 'mkrn3 gene'/de OR 'makorin ring finger protein 3'/de OR 'magel2 gene'/de OR 'magel2 protein'/de OR 'necdin'/de OR 'small nuclear ribonucleoprotein polypeptide n'/de OR 'snrpn gene'/de OR 'snrpn protein'/de OR 'e6 associated protein'/de OR ((Prader* NEAR/3 Willi*) OR PraderWilli OR mkrn3 OR makorin-3 OR znf127 OR D15S9 OR RNF63 OR EC-2.3.2.27 OR ZFP127 OR CPPB2 OR magel2 OR magel-2 OR ((makorin) NEAR/3 (ring) NEAR/3 (3)) OR ((zinc) NEAR/3 (finger) NEAR/3 (127)) OR ((ring) NEAR/3 (finger) NEAR/3 (63)) OR ((MAGE OR melanoma) NEAR/3 (family) NEAR/3 (L2)) OR ((necdin-like OR MAGE-like) NEAR/3 (protein) NEAR/3 (1 OR 2)) OR NDNL1 OR MAGE-Like-2 OR SHFYNG OR PWLS OR NM15 OR (NDN NOT nonclassic-differentiation-number*) OR necdin* OR HsT16328 OR PWCR OR C15orf2 OR NPAP1 OR NPAP-1 OR Nuclear-Pore-Associated-Protein-1 OR ((chromosome-15) NEAR/3 (open-reading-frame* OR ORF*) NEAR/3 (2)) OR SNURF-SNRPN* OR SNRPN* OR Small-Nuclear-Ribonucleoprotein-Polypeptide-N* OR Small-Nuclear-Ribonucleoprotein-Associated-Protein-N* OR Tissue-Specific-Splicing-Protein* OR Sm-Protein-D OR SM-Protein-N OR HCERN3 OR (((Sm-N OR SMN OR Sm-D OR PET1 OR BEY OR PED) AND (gene OR genes OR genetic OR genome* OR genomic)) NOT (sec*-malign*-neoplas* OR survival-motor-neuro*)) OR Small-Nucl*-Ribonucleoprot*-N OR RT-LI OR PWCR OR SNORD107 OR SNORD-107 OR (("C/D" OR CD) NEAR/3 (box*) NEAR/3 (107 OR 64 OR 109A OR 116 OR 115 OR 109B)) OR HBII-436 OR RF01164 OR SNORD64 OR HBII-13 OR RF00570 OR SNORD109A OR HBII-438A OR RF01278 OR SNORD116 OR HBII-85 OR PWCR1 OR SNORD115 OR HBII-52 OR RNHBII52 OR RF00105 OR SNORD109B OR HBII-438B OR RF01278 OR KIAA1899):ab,ti,kw) **AND** ('urinary tract function'/exp OR 'urinary tract disease'/exp OR 'urinary tract'/exp OR 'urologic examination'/exp OR 'dipeptidyl carboxypeptidase inhibitor'/exp OR 'angiotensin converting enzyme 2'/de OR 'angiotensin II antagonist'/de OR 'creatinine'/de OR 'hemodialysis'/exp OR 'peritoneal dialysis'/exp OR 'plasma clearance'/de OR 'diuretic agent'/exp OR 'kidney transplantation'/exp OR 'cholesterol'/exp OR 'renal replacement therapy'/de OR 'renin angiotensin aldosterone system'/de OR 'abnormal blood pressure'/exp OR 'urine'/de OR (kidney* OR renal* OR renovasc* OR nephro* OR nefro* OR ureter* OR urethra* OR paraurethr* OR urin* OR nephritis* OR proteinuri* OR albuminuria* OR microalbuminuria* OR macroalbuminuria* OR (ACE NEAR/3 (inhibitor* OR blocker* OR antagonist*)) OR Sulfhydryl-containing-agent* OR Alacepril* OR Captopril* OR Zefnopril* OR Dicarboxylate-containing-agent* OR Enalapril* OR Vasotec* OR Renitec* OR Berlipril* OR Enap* OR Ramipril* OR Altace* OR Prilace* OR Ramace* OR Ramiwin* OR Triatec* OR Ramitac* OR Quinapril* OR Accupril* OR Perindopril* OR Coversyl* OR Aceon* OR Perindo* OR Lisinopril* OR Listril* OR Lopril* OR Novatec* OR Prinivil* OR Zestril* OR Lisidigal* OR Benazepril* OR Lotensin* OR Imidapril* OR Tanatril* OR Trandolapril* OR Mavik* OR Odrik* OR Gopten* OR Cilazapril* OR Inhibace* OR Phosphonate-containing-agent* OR Fosinopril* OR Fositen* OR Monopril* OR Angiotensinconverting-enzyme* OR Angiotensin-converting-enzyme* OR ((angiotensin*) NEAR/3 (2 OR ii) NEAR/3 (inhibitor* OR blocker* OR antagonist*)) OR Azilsartan* OR Edarbi* OR Candesartan* OR Atacand* OR Irbesartan* OR Avapro* OR Losartan* OR Cozaar* OR Olmesartan* OR Benicar* OR Telmisartan* OR Micardis* OR Valsartan* OR Diovan* OR Teveten* OR eprosartan* OR Edarbi* OR Azilsartan-medoxomil* OR Prexxarta OR renin-angiotensin* OR glomerul* OR GFR OR creatinin* OR dialysis* OR MDRD OR CKD-EPI OR diuretic* OR cholest* OR hypercholest* OR spironolactone* OR hypospadia* OR epispadia* OR urolog* OR blood-press* OR hypertens* OR hypotens*):ab,ti,kw) *NOT ([Conference Abstract]/lim AND [1800-2017]/py)* |
| --- |
| **Medline (Ovid)**  (Prader-Willi Syndrome/ OR ((Prader* ADJ3 Willi*) OR PraderWilli OR mkrn3 OR makorin-3 OR znf127 OR D15S9 OR RNF63 OR "EC-2.3.2.27" OR ZFP127 OR CPPB2 OR magel2 OR magel-2 OR ((makorin) ADJ3 (ring) ADJ3 ("3")) OR ((zinc) ADJ3 (finger) ADJ3 ("127")) OR ((ring) ADJ3 (finger) ADJ3 ("63")) OR ((MAGE OR melanoma) ADJ3 (family) ADJ3 (L2)) OR ((necdin-like OR MAGE-like) ADJ3 (protein) ADJ3 (1 OR "2")) OR NDNL1 OR MAGE-Like-2 OR SHFYNG OR PWLS OR NM15 OR (NDN NOT nonclassic-differentiation-number*) OR necdin* OR HsT16328 OR PWCR OR C15orf2 OR NPAP1 OR NPAP-1 OR Nuclear-Pore-Associated-Protein-1 OR ((chromosome-15) ADJ3 (open-reading-frame* OR ORF*) ADJ3 ("2")) OR SNURF-SNRPN* OR SNRPN* OR Small-Nuclear-Ribonucleoprotein-Polypeptide-N* OR Small-Nuclear-Ribonucleoprotein-Associated-Protein-N* OR Tissue-Specific-Splicing-Protein* OR Sm-Protein-D OR SM-Protein-N OR HCERN3 OR (((Sm-N OR SMN OR Sm-D OR PET1 OR BEY OR PED) AND (gene OR genes OR genetic OR genome* OR genomic)) NOT (sec*-malign*-neoplas* OR survival-motor-neuro*)) OR Small-Nucl*-Ribonucleoprot*-N OR RT-LI OR PWCR OR SNORD107 OR SNORD-107 OR (("C/D" OR CD) ADJ3 (box*) ADJ3 (107 OR 64 OR 109A OR 116 OR 115 OR 109B)) OR HBII-436 OR RF01164 OR SNORD64 OR HBII-13 OR RF00570 OR SNORD109A OR HBII-438A OR RF01278 OR SNORD116 OR HBII-85 OR PWCR1 OR SNORD115 OR HBII-52 OR RNHBII52 OR RF00105 OR SNORD109B OR HBII-438B OR RF01278 OR KIAA1899).ab,ti,kf.) **AND** (exp Urologic Diseases/ OR exp Diagnostic Techniques, Urological/ OR exp Urinary Tract/ OR exp Urinary Tract Physiological Phenomena/ OR exp Angiotensin-Converting Enzyme Inhibitors/ OR Creatinine/ OR exp Renal Replacement Therapy/ OR exp Diuretics/ OR exp Cholesterol/ OR Renin-Angiotensin System/ OR Hypertension/ OR exp Hypotension/ OR Urine/ OR urine.fs. OR (kidney* OR renal* OR renovasc* OR nephro* OR nefro* OR ureter* OR urethra* OR paraurethr* OR urin* OR nephritis* OR proteinuri* OR albuminuria* OR microalbuminuria* OR macroalbuminuria* OR (ACE ADJ3 (inhibitor* OR blocker* OR antagonist*)) OR Sulfhydryl-containing-agent* OR Alacepril* OR Captopril* OR Zefnopril* OR Dicarboxylate-containing-agent* OR Enalapril* OR Vasotec* OR Renitec* OR Berlipril* OR Enap* OR Ramipril* OR Altace* OR Prilace* OR Ramace* OR Ramiwin* OR Triatec* OR Ramitac* OR Quinapril* OR Accupril* OR Perindopril* OR Coversyl* OR Aceon* OR Perindo* OR Lisinopril* OR Listril* OR Lopril* OR Novatec* OR Prinivil* OR Zestril* OR Lisidigal* OR Benazepril* OR Lotensin* OR Imidapril* OR Tanatril* OR Trandolapril* OR Mavik* OR Odrik* OR Gopten* OR Cilazapril* OR Inhibace* OR Phosphonate-containing-agent* OR Fosinopril* OR Fositen* OR Monopril* OR Angiotensinconverting-enzyme* OR Angiotensin-converting-enzyme* OR ((angiotensin*) ADJ3 ("2" OR ii) ADJ3 (inhibitor* OR blocker* OR antagonist*)) OR Azilsartan* OR Edarbi* OR Candesartan* OR Atacand* OR Irbesartan* OR Avapro* OR Losartan* OR Cozaar* OR Olmesartan* OR Benicar* OR Telmisartan* OR Micardis* OR Valsartan* OR Diovan* OR Teveten* OR eprosartan* OR Edarbi* OR Azilsartan-medoxomil* OR Prexxarta OR renin-angiotensin* OR glomerul* OR GFR OR creatinin* OR dialysis* OR MDRD OR CKD-EPI OR diuretic* OR cholest* OR hypercholest* OR spironolactone* OR hypospadia* OR epispadia* OR urolog* OR blood-press* OR hypertens*).ab,ti,kf.) *NOT (news OR congres* OR abstract* OR book* OR chapter* OR dissertation abstract*).pt.* |
| **Web of Science Core Collection**  TS=((((Prader* NEAR/2 Willi*) OR PraderWilli OR mkrn3 OR makorin-3 OR znf127 OR D15S9 OR RNF63 OR EC-2.3.2.27 OR ZFP127 OR CPPB2 OR magel2 OR magel-2 OR ((makorin) NEAR/2 (ring) NEAR/2 (3)) OR ((zinc) NEAR/2 (finger) NEAR/2 (127)) OR ((ring) NEAR/2 (finger) NEAR/2 (63)) OR ((MAGE OR melanoma) NEAR/2 (family) NEAR/2 (L2)) OR ((necdin-like OR MAGE-like) NEAR/2 (protein) NEAR/2 (1 OR 2)) OR NDNL1 OR MAGE-Like-2 OR SHFYNG OR PWLS OR NM15 OR (NDN NOT nonclassic-differentiation-number*) OR necdin* OR HsT16328 OR PWCR OR C15orf2 OR NPAP1 OR NPAP-1 OR Nuclear-Pore-Associated-Protein-1 OR ((chromosome-15) NEAR/2 (open-reading-frame* OR ORF*) NEAR/2 (2)) OR SNURF-SNRPN* OR SNRPN* OR Small-Nuclear-Ribonucleoprotein-Polypeptide-N* OR Small-Nuclear-Ribonucleoprotein-Associated-Protein-N* OR Tissue-Specific-Splicing-Protein* OR Sm-Protein-D OR SM-Protein-N OR HCERN3 OR (((Sm-N OR SMN OR Sm-D OR PET1 OR BEY OR PED) AND (gene OR genes OR genetic OR genome* OR genomic)) NOT (sec* NEXT malign* NEXT neoplas* OR survival-motor-neuro*)) OR Small-Nucl* NEXT Ribonucleoprot* NEXT N OR RT-LI OR PWCR OR SNORD107 OR SNORD-107 OR (("C/D" OR CD) NEAR/2 (box*) NEAR/2 (107 OR 64 OR 109A OR 116 OR 115 OR 109B)) OR HBII-436 OR RF01164 OR SNORD64 OR HBII-13 OR RF00570 OR SNORD109A OR HBII-438A OR RF01278 OR SNORD116 OR HBII-85 OR PWCR1 OR SNORD115 OR HBII-52 OR RNHBII52 OR RF00105 OR SNORD109B OR HBII-438B OR RF01278 OR KIAA1899)) **AND** ((kidney* OR renal* OR renovasc* OR nephro* OR nefro* OR ureter* OR urethra* OR paraurethr* OR urin* OR nephritis* OR proteinuri* OR albuminuria* OR microalbuminuria* OR macroalbuminuria* OR (ACE NEAR/2 (inhibitor* OR blocker* OR antagonist*)) OR Sulfhydryl-containing-agent* OR Alacepril* OR Captopril* OR Zefnopril* OR Dicarboxylate-containing-agent* OR Enalapril* OR Vasotec* OR Renitec* OR Berlipril* OR Enap* OR Ramipril* OR Altace* OR Prilace* OR Ramace* OR Ramiwin* OR Triatec* OR Ramitac* OR Quinapril* OR Accupril* OR Perindopril* OR Coversyl* OR Aceon* OR Perindo* OR Lisinopril* OR Listril* OR Lopril* OR Novatec* OR Prinivil* OR Zestril* OR Lisidigal* OR Benazepril* OR Lotensin* OR Imidapril* OR Tanatril* OR Trandolapril* OR Mavik* OR Odrik* OR Gopten* OR Cilazapril* OR Inhibace* OR Phosphonate-containing-agent* OR Fosinopril* OR Fositen* OR Monopril* OR Angiotensinconverting-enzyme* OR Angiotensin-converting-enzyme* OR ((angiotensin*) NEAR/2 (2 OR ii) NEAR/2 (inhibitor* OR blocker* OR antagonist*)) OR Azilsartan* OR Edarbi* OR Candesartan* OR Atacand* OR Irbesartan* OR Avapro* OR Losartan* OR Cozaar* OR Olmesartan* OR Benicar* OR Telmisartan* OR Micardis* OR Valsartan* OR Diovan* OR Teveten* OR eprosartan* OR Edarbi* OR Azilsartan-medoxomil* OR Prexxarta OR renin-angiotensin* OR glomerul* OR GFR OR creatinin* OR dialysis* OR MDRD OR CKD-EPI OR diuretic* OR cholest* OR hypercholest* OR spironolactone* OR hypospadia* OR epispadia* OR urolog* OR blood-press* OR hypertens* OR hypotens*))) *AND DT=(Article OR Review)* |
| **Cochrane Central Register of Controlled Trials**  (((Prader* NEAR/3 Willi*) OR PraderWilli OR mkrn3 OR makorin-3 OR znf127 OR D15S9 OR RNF63 OR EC-2.3.2.27 OR ZFP127 OR CPPB2 OR magel2 OR magel-2 OR ((makorin) NEAR/3 (ring) NEAR/3 (3)) OR ((zinc) NEAR/3 (finger) NEAR/3 (127)) OR ((ring) NEAR/3 (finger) NEAR/3 (63)) OR ((MAGE OR melanoma) NEAR/3 (family) NEAR/3 (L2)) OR ((necdin-like OR MAGE-like) NEAR/3 (protein) NEAR/3 (1 OR 2)) OR NDNL1 OR MAGE-Like-2 OR SHFYNG OR PWLS OR NM15 OR (NDN NOT nonclassic-differentiation-number*) OR necdin* OR HsT16328 OR PWCR OR C15orf2 OR NPAP1 OR NPAP-1 OR Nuclear-Pore-Associated-Protein-1 OR ((chromosome-15) NEAR/3 (open-reading-frame* OR ORF*) NEAR/3 (2)) OR SNURF-SNRPN* OR SNRPN* OR Small-Nuclear-Ribonucleoprotein-Polypeptide-N* OR Small-Nuclear-Ribonucleoprotein-Associated-Protein-N* OR Tissue-Specific-Splicing-Protein* OR Sm-Protein-D OR SM-Protein-N OR HCERN3 OR (((Sm-N OR SMN OR Sm-D OR PET1 OR BEY OR PED) AND (gene OR genes OR genetic OR genome* OR genomic)) NOT (sec* NEXT malign* NEXT neoplas* OR survival-motor-neuro*)) OR Small-Nucl* NEXT Ribonucleoprot* NEXT N OR RT-LI OR PWCR OR SNORD107 OR SNORD-107 OR (("C/D" OR CD) NEAR/3 (box*) NEAR/3 (107 OR 64 OR 109A OR 116 OR 115 OR 109B)) OR HBII-436 OR RF01164 OR SNORD64 OR HBII-13 OR RF00570 OR SNORD109A OR HBII-438A OR RF01278 OR SNORD116 OR HBII-85 OR PWCR1 OR SNORD115 OR HBII-52 OR RNHBII52 OR RF00105 OR SNORD109B OR HBII-438B OR RF01278 OR KIAA1899):ab,ti,kw) **AND** ((kidney* OR renal* OR renovasc* OR nephro* OR nefro* OR ureter* OR urethra* OR paraurethr* OR urin* OR nephritis* OR proteinuri* OR albuminuria* OR microalbuminuria* OR macroalbuminuria* OR (ACE NEAR/3 (inhibitor* OR blocker* OR antagonist*)) OR Sulfhydryl-containing-agent* OR Alacepril* OR Captopril* OR Zefnopril* OR Dicarboxylate-containing-agent* OR Enalapril* OR Vasotec* OR Renitec* OR Berlipril* OR Enap* OR Ramipril* OR Altace* OR Prilace* OR Ramace* OR Ramiwin* OR Triatec* OR Ramitac* OR Quinapril* OR Accupril* OR Perindopril* OR Coversyl* OR Aceon* OR Perindo* OR Lisinopril* OR Listril* OR Lopril* OR Novatec* OR Prinivil* OR Zestril* OR Lisidigal* OR Benazepril* OR Lotensin* OR Imidapril* OR Tanatril* OR Trandolapril* OR Mavik* OR Odrik* OR Gopten* OR Cilazapril* OR Inhibace* OR Phosphonate-containing-agent* OR Fosinopril* OR Fositen* OR Monopril* OR Angiotensinconverting-enzyme* OR Angiotensin-converting-enzyme* OR ((angiotensin*) NEAR/3 (2 OR ii) NEAR/3 (inhibitor* OR blocker* OR antagonist*)) OR Azilsartan* OR Edarbi* OR Candesartan* OR Atacand* OR Irbesartan* OR Avapro* OR Losartan* OR Cozaar* OR Olmesartan* OR Benicar* OR Telmisartan* OR Micardis* OR Valsartan* OR Diovan* OR Teveten* OR eprosartan* OR Edarbi* OR Azilsartan-medoxomil* OR Prexxarta OR renin-angiotensin* OR glomerul* OR GFR OR creatinin* OR dialysis* OR MDRD OR CKD-EPI OR diuretic* OR cholest* OR hypercholest* OR spironolactone* OR hypospadia* OR epispadia* OR urolog* OR blood-press* OR hypertens* OR hypotens*):ab,ti,kw) |
| **Google Scholar**  ‘Prader Willi’ kidney\|renal\|renovascular\|ureter\|urethra\|urinal\|urine\|proteinuria\|albuminuria\|‘ACE\|angiotensin inhibitor\|blocker\|antagonist’\|glomeruli\|creatinine\|dialysis\|diuretics\|cholesterol\|hypertension |
